# Pre-treatment and post-treatment nasopharyngeal carcinoma imaging: imaging updates, pearls and pitfalls

**DOI:** 10.1007/s00234-025-03596-z

**Published:** 2025-04-11

**Authors:** Kwok Yan Li, Hoi Ming Kwok, Nin Yuan Pan, Lik Fai Cheng, Ka Fai Johnny Ma

**Affiliations:** 1 Department of Diagnostic and Interventional Radiology, Princess Margaret Hospital, Hong Kong SAR, China; 2 Department of Imaging and Interventional Radiology, Chinese University of Hong Kong, Hong Kong SAR, China

**Keywords:** Nasopharyngeal carcinoma, Imaging, Computed tomography, Positron emission tomography, Magnetic resonance imaging, Diffusion-weighted imaging

## Abstract

****Purpose**:**

Nasopharyngeal carcinoma (NPC) is endemic in Southeast Asia, requiring precise imaging for personalized treatment. This review highlights key imaging challenges and updates from recent literature, emphasizing findings that impact oncological management.

****Methods**:**

We discuss common and uncommon clinical entities, detailing salient imaging features and diagnostic distinctions to aid accurate interpretation.

****Results**:**

In the pre-treatment setting, leveraging the characteristic MR signals and spread patterns of NPC aids in defining the tumor volume for accurate staging and radiotherapy contouring. Key diagnostic challenges include differentiating tumor from benign hyperplasia, skull base osteomyelitis, and other skull base tumors. Perineural tumor spread, radiological extranodal extension and nodal necrosis further refine primary tumor and nodal assessment. In the post-treatment setting, the key question is whether tumor recurrence exists. Diagnostic challenges involve distinguishing tumor recurrence from scar tissue, post-radiation nasopharyngeal necrosis, or hypertrophied cervical ganglia. For recurrences, endoscopic nasopharyngectomy has emerged as the preferred approach over open surgery or re-irradiation. The text highlights characteristic post-treatment appearances and emphasizes recognizing these patterns to avoid misinterpretation and guide appropriate management.

****Conclusion**:**

Imaging plays a pivotal role in NPC precision oncology. Mastering imaging pearls and pitfalls empowers radiologists to provide clinicians with reliable, actionable guidance.

## Introduction

Nasopharyngeal carcinoma (NPC) is a prevalent tumor in Southeast Asia, especially in Southern China. It is related to Ebstein-Barr virus (EBV) infection. Its incidence is more than 20 per 100,000 person-years in endemic regions, while less than 1 per 100,000 person-years in most parts of the world [1]. Evolving evidence in pre- and post-treatment NPC urges increasingly personalized and precise oncological treatment, demanding expertise and close communication between radiologists and oncologists. This review provides insights into the key aspects and challenges in NPC imaging with updates from recently published literature, emphasizing those with oncological management impact (Table 1). In the pre-treatment setting, the prime objective is to define the tumor volume accurately for staging and subsequent radiotherapy contouring. In the post-treatment setting, the key question is whether tumor recurrence exists.

**Table 1:** Summary of the pearls and pitfalls in NPC imaging that impact oncological decision making

	Pearls and pitfalls	Management impact
Pre-treatment	1. Radiologists should define and map the tumor volume besides accurate staging. The extent of involvement needs to be communicated to the radiation oncologists 2. The nasopharynx is an important review area in brain imaging. Not every soft tissue in the nasopharynx is a tumor 3. Perineural spread (PNS) in NPC causes tumor spread to distant areas beyond the confinement of the primary tumor 4. Radiologists should be aware of nodal features beyond nodal staging, such as necrosis and radiological extranodal extension (ENE) 5. Various tumor mimics exist in the nasopharynx varying from benign to malignant conditions	1. Accurate primary tumor delineation forms the basis of precision radiotherapy in achieving the most dose-effective local or locoregional control 2. Adenoidal hyperplasia can mimic a tumor or coexist with a tumor. Normal variants in the nasopharynx require no treatment. Identification of tumor prompts further targeted biopsy and formal staging 3. Underdetection of PNS leads to missing radiation target and potential local recurrence 4. Nodal necrosis and radiological ENE are independent poor prognosticators. Presence of radiological ENE may require special radiotherapy contouring technique 5. Accurate diagnosis of tumor mimics avoids mis-management
Post-treatment	1. Recurrent tumors most commonly are in-field failure and a distinction has to be made with various post-treatment changes. Additional challenges are related to delayed tumor regression and time lag between histological and radiological regression 2. Post-radiation nasopharyngeal necrosis is a life-threatening condition mimicking tumor recurrence 3. Not every enlarging retropharyngeal mass is a sign of malignancy. Hypertrophied superior cervical ganglia can be observed in post-irradiation setting mimicking a recurrent retropharyngeal node 4. Skull base marrow signal change after radiotherapy does not always equal recurrent or residual tumor 5. Familiarity with the expected imaging appearance of nasopharyngectomy is essential for accurate image interpretation of post-operative changes versus recurrent tumors	1. Presence of recurrent tumor requires additional investigations and salvage therapy. Residual pathological neck nodes necessitate neck dissection. Absence of residual or recurrent disease should direct clinical, endoscopic and imaging surveillance 2. Correct diagnosis of post-radiation nasopharyngeal necrosis avoids unnecessary investigation and repeated biopsies. Massive bleeding from carotid artery erosion leads to mortality 3. Hypertrophied superior cervical ganglia itself requires no further management. Recurrent retropharyngeal nodes require complete re-staging and further investigation 4. Skull base marrow signal change can persist for years after radiotherapy without a viable tumor. A distinction has to be made for this versus recurrent tumor which requires further management 5. Expected postoperative change requires imaging surveillance, while recurrent tumors require further investigation and salvage therapy

## Pre-treatment imaging

1. *Interpretation of NPC MRI goes beyond local staging. Accurate primary tumor delineation forms the basis of precision radiotherapy in achieving the most dose-effective local or locoregional control. Radiologists need to recognize the characteristic tumor signal and have a tailored search pattern for the characteristic pattern of tumor spread.*

Nowadays, NPC is primarily treated by radiotherapy via intensity-modulated radiotherapy (IMRT) with or without chemotherapy as the standard of care. Successful curative radiotherapy aims to maximize the primary tumor's loco-regional control and minimize treatment-related complications to the organ at risk (OAR). This relies on robust imaging techniques that accurately map the tumor, interpretation by subspecialized head and neck radiologists, and effective communication of the image findings with its management and prognostication information to the radiation oncologists.

MRI is the primary modality not only for local staging but also for radiotherapy planning due to its superior soft tissue visualization compared to 18F-fluorodeoxyglucose positron emission tomography/computed tomography (FDG-PET/CT) and computed tomography (CT) [2]. Fusion of MR images with planning CT images enables radiation oncologists to carry out precise radiotherapy planning. A state-of-the-art MR protocol for NPC imaging comprises both two-dimensional and three-dimensional sequences [2]. For two-dimensional sequences, axial fat-suppressed T2-weighted, T1-weighted pre- and post-contrast, and diffusion-weighted images (DWI) (with b-value at least 800 s/mm^2^) should be performed with a scanned range from above the skull base to C3 [2]. Coronal T1-weighted pre- and post-contrast, as well as sagittal T1-weighted post-contrast sequences, could also be performed. Dedicated neck images of axial T1-weighted post-contrast sequence from C3 to the level below the suprasternal notch should be included for screening lower neck and supraclavicular nodes. The three-dimensional sequence can be obtained with a fat-suppressed T1-weighted sequence in the coronal plane with multiplanar reformat [2]. DWI with non-echoplanar imaging (non-EPI) or multishot echoplanar imaging (msEPI) techniques should be performed instead of single-shot echoplanar imaging (ssEPI) as they give less susceptibility-related distortion in the skull base. The examination should be performed on a 1.5-T or preferably a 3.0-T MRI with the use of a 64-channel head and neck coil, to achieve a better signal-to-noise ratio, spatial resolution, and a shorter scanning time.

Accurate target delineation for gross tumor volume (GTV) contouring is challenging given the complex locoregional anatomy and the intricate relation between the tumor and multiple OARs [3]. The clinical target volume (CTV) covers possible microscopic disease around the tumor. Its contouring relies on accurate and precise GTV contouring on both the primary tumor and nodal metastases. The traditional approach of "5 + 5" recommendation of GTV expansion was recommended from pathological evidence of head and neck squamous cell carcinoma, referring to a 5 mm volumetric expansion from the GTV as high-risk CTV, and another 5 mm expansion from the high risk CTV as low-risk CTV [3]. A tighter margin of 1–2 mm is recommended for primary tumor encroaching critical neural OARs (e.g., optic nerves, optic chiasm, brainstem, etc.) [3]. There has been ongoing research and advancement towards a more individualized approach to CTV contouring based on tumor extent and spread pattern, including the sparing of contralateral structures in unilateral disease and reduction in CTV upon response to induction chemotherapy [3]. Besides the primary tumor, CTV delineation of regional lymphatics moves towards the refinement of delineation boundaries in nodal level according to nodal spread pattern with the omission of lower neck irradiation in the uninvolved neck [3].

Radiologists need to have a tailored search pattern for nasopharyngeal tumor, given that the primary tumor spreads in a highly predictable and orderly fashion [4–7]. The same principle also applies to the contouring of the CTV by radiation oncologists [7]. The primary tumor spreads via a stepwise fashion from the high-risk anatomical region around the nasopharynx (e.g., parapharyngeal space, prevertebral space, pterygoid process, and basisphenoid) to the more distal regions [4, 5]. Besides, the nodal metastases of NPC also follow an orderly fashion, and skip metastasis only occurs in 0.5–7.9% of cases [8]. Both the level II and retropharyngeal nodes can be echelon nodes with involvement in 70% and 69% of the cases, respectively [3, 8]. In interpreting tumoral involvement within the delicate head and neck structures, emphasis should be placed on the use of T1-weighted non-fat-suppressed images and the recognition of the characteristic tumoral signal on T2-weighted images. Careful inspection of structural asymmetry and soft tissue replacement of the fat signal in the skull base on a non-fat-suppressed T1-weighted image may be the first clue to guide radiologists to look for tumor involvement. NPC has a characteristic intermediate T2-weighted signal. It demonstrates intermediate enhancement after gadolinium injection [2]. This can serve as confirmation of tumoral involvement when both the structural asymmetry and the signal characteristic fit the pattern of tumor spread. Radiologists are adept in utilizing multisequence MRI, including DWI, and multiplanar reformats to navigate between the complex anatomy to map out the full extent of the tumor. NPC shows restricted diffusion on DWI due to hypercellularity, with a mean apparent diffusion coefficient (ADC) value of 871.7 ± 84.4 × 10⁻⁶ mm^2^/s [9]. The distinctive high signal of the tumor on a high b-value DWI image and low signal in the ADC map relative to adjacent soft tissue structures can provide invaluable information on tumor margin for accurate tumor volume delineation [10]. A certain degree of peritumoral inflammation is commonly observed when NPC spreads to the parapharyngeal space adjacent to the masticator muscles, or the skull base. These can be differentiated from tumor involvement by a higher T2-weighted signal and a higher degree of enhancement, without associated restricted diffusion. This distinction is important in radiotherapy contouring.

2. *Recognition of normal variants in the nasopharynx avoids misdiagnosis, over-investigation, and over-treatment. Radiologists should be aware of the coexistence of adenoidal hyperplasia and tumors.*

The distinction of adenoidal hyperplasia from NPC on MRI is important, as the former is a benign condition that requires no further investigation. It is a common clinical encounter when patients are referred for MRI assessment of the non-specific nasopharyngeal soft tissue thickening in other imaging modalities, such as CT or PET/CT, or in MRI dedicated to the brain or other neck regions. With the advent of successful plasma Epstein-Barr virus DNA (EBV-DNA) screening for early-stage NPC, asymptomatic patients are referred for a screening MRI when they have a normal endoscopic exam or indeterminate soft tissue bulge in the nasopharynx [2, 11, 12]. In these instances, the distinction between normal variants and tumors in the nasopharynx becomes crucial. Despite the international guideline recommendation for endoscopic biopsy of the nasopharynx to establish the definitive diagnosis of NPC, about 10%−30% of NPC are missed at initial endoscopy due to small size, submucosal location, coexistent hyperplasia, and anatomical difficulty in accessing the lateral pharyngeal recess [2, 12–14]. A meta-analysis with 7 out of 9 studies using contrast-enhanced MR protocols shows that MRI has a very high pooled sensitivity of 98.1% (95% CI 95.2–99.3%) and specificity of 91.7% (95% CI 88.3–94.2%) for the detection of NPC [13]. MRI is also more sensitive compared with endoscopy (91.2% versus 76.5%) in the detection of early NPC in a prospective study in asymptomatic individuals with elevated plasma EBV-DNA [12]. A recent prospective study using a quick and short contrast-free MRI screening protocol for NPC detection in EBV-positive individuals shows a high negative predictive value of 99.4%, complementing endoscopy and endoscopic-guided biopsy in the screening setting [15]. Radiologists should be equipped with the essential knowledge for making this distinction and be aware of the possible challenge of a coexisting tumor and adenoidal hyperplasia.

**Fig. 1:**
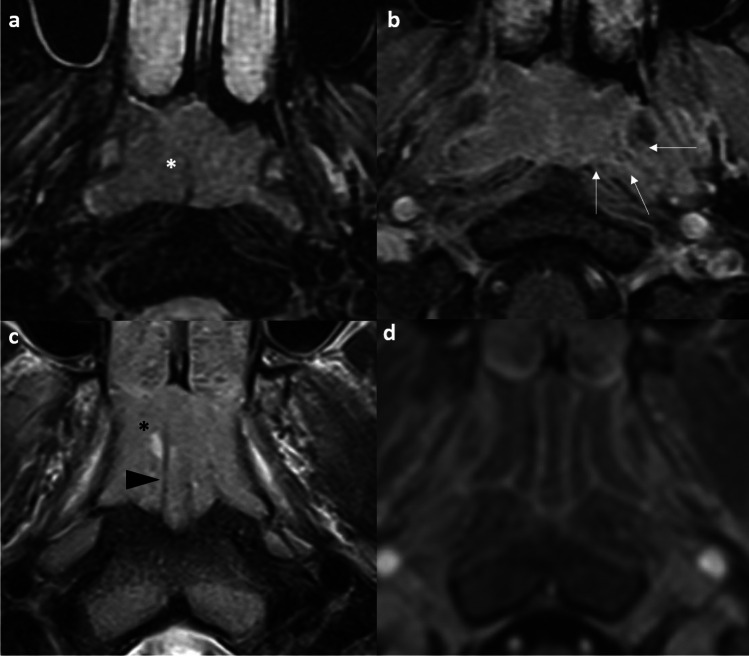
Distinction between nasopharyngeal tumor (a & b) and adenoidal hyperplasia (c & d). Axial T2-weighted, fat-suppressed image shows intermediate signal slightly asymmetrical soft tissue occupying the bilateral nasopharynx with absence of the adenoidal septa (asterisk) (**a**). Axial T1-weighted post-contrast image with fat suppression shows moderate enhancement of the bilateral nasopharyngeal soft tissue mass with focal loss of the deep white mucosal line in the left lateral and posterior wall (arrows) (**b**). Biopsy confirmed nasopharyngeal carcinoma. Axial T2-weighted, fat suppressed image shows the symmetrical soft tissue in the bilateral nasopharynx containing cystic focus (asterisk) and preservation of hypointense adenoidal septa (arrowhead) (**c**). Axial T1-weighted post-contrast image with fat suppression shows symmetrical hypo-enhancing soft tissue with a striated appearance and preservation of deep mucosal white line (**d**). Findings are consistent with adenoidal hyperplasia

Adenoidal hyperplasia is common and physiological in children between the ages of 6 and 10 years and then regresses by puberty. However, it is also possible to encounter nasopharyngeal tissue proliferation in adults, especially smokers [16, 17]. The typical MR appearance includes stripes in adenoid bulges on post-contrast T1-weighted images, preservation of deep white mucosal lines, and structural symmetry [18, 19]. Other findings such as cysts (41%) and Tornwaldt cysts (4%) can also be seen [18]. Subsequent work by King et al. suggested several distinctive MRI imaging features to suggest NPC, including a significantly greater volume, size asymmetry, signal asymmetry, focal loss of the deep mucosal white line, and absence/ distortion of the adenoidal septa [19] (Fig. 1). The size asymmetry was the most accurate criterion (89.5%) for such distinction [19].

**Fig. 2:**
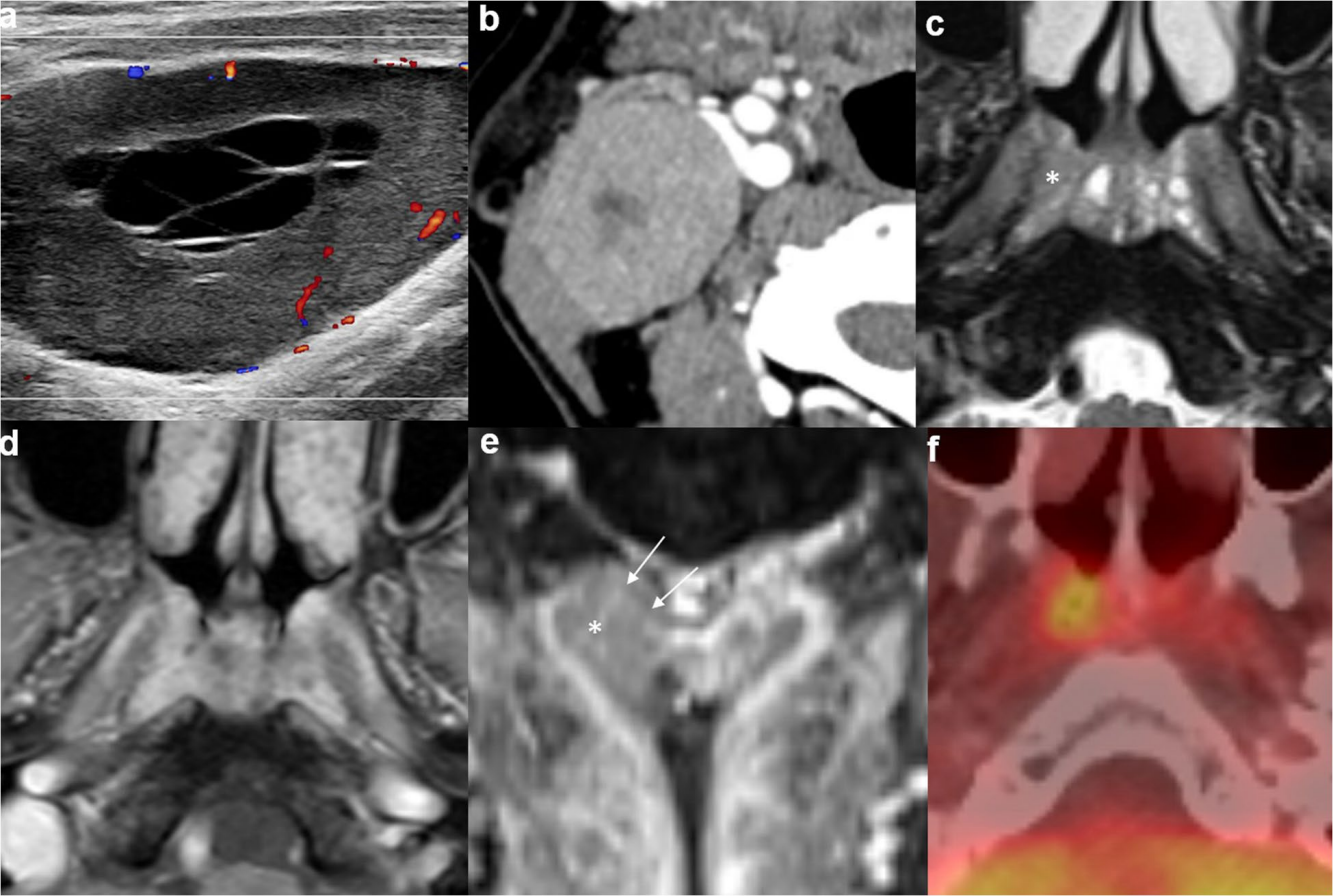
Coexistence of nasopharyngeal tumor and lymphoid hyperplasia in a 70-year-old man referred for an enlarging right neck mass. Doppler ultrasound of the right neck shows a hypoechoic mass with increased peripheral vascular flow and a central anechoic cystic area with septations (**a**). Axial contrast CT of the neck shows an enlarged roundish right level II node with central hypoenhancing area suggestive of necrosis (**b**). Prominent soft tissue density in the nasopharynx was noted on the CT (not shown). Endoscopy found a right nasopharyngeal mass and biopsy was proven to be an undifferentiated carcinoma. Axial T2-weighted image shows soft tissue thickening of the bilateral nasopharynx. Small cysts are seen involving the bilateral nasopharynx, more on the left side. Minimal intermediate T2-weighted soft tissue thickening is seen at the lateral wall of right nasopharynx (asterisk) (**c**). The pharyngobasilar fascia is intact. Axial post-contrast T1-weighted, fat-suppressed image shows only non-specific moderately enhancing soft tissue thickening of the bilateral nasopharynx with intact deep mucosal white line (**d**). Coronal post-contrast, fat-suppressed T1-weighted image depicts a moderately enhancing mass in the right nasopharynx (asterisk) with focal loss of the deep mucosal white line at the roof (arrows) (**e**). Axial fusion FDG-PET/CT depicts the hyper-metabolic right nasopharyngeal mass and the hypermetabolic right level II node (not shown). Otherwise, no hypermetabolic distant metastasis (**f**). Radiological staging was T1N1M0 and the patient received radical radiotherapy. This case highlights three diagnostic pearls. First, the presence of benign lymphoid hyperplasia and tumor are not mutually exclusive. Second, the use of multiplanar reformat should be routinely performed for evaluation of the nasopharynx in order to avoid missing small tumors due to partial volume effect. Third, level II nodal metastasis can occur without involvement of the retropharyngeal node, especially for laterally located tumors in the nasopharynx

It can be challenging to distinguish a small T1 tumor or a fairly symmetric-looking tumor from adenoidal hyperplasia. In general, the above rules still apply in making the distinction. Identification of a small T1 tumor guides the site and depth of biopsy for endoscopic surgeons [2]. Furthermore, adenoidal hyperplasia and NPC can coexist [2, 20] (Fig. 2), highlighting the danger of tumor underdetection from satisfaction of search. The identification of coexisting adenoidal hyperplasia allows precise delineation of GTV during radiotherapy planning and anticipation of a different post-treatment imaging appearance compared to the main tumor bulk on follow-up imaging, avoiding unnecessary repeated biopsy [20].

3. *Perineural spread forms a tumor highway in NPC, potentially causing missing radiation targets and risk of residual tumor.*

Perineural tumor growth is a well-recognized clinicopathological entity in the head and neck that is associated with poorer prognosis and an increased risk of locoregional recurrence [21]. It encompasses perineural invasion (PNI) and perineural spread (PNS). PNI is a histological diagnosis of tumor cell infiltration into nerves at the original tumor sites, which cannot be radiologically detected. PNS refers to macroscopic tumor involvement along a nerve extending away from the primary tumor, evading from the effective radiation field. This can be radiologically detected [21–23].

The incidence of PNS or PNI has been reported to be 27–82% in head and neck squamous cell carcinomas [21]. In NPC, Liu et al. evaluated MRI-detected PNI, together with PNS, giving a total incidence of approximately 39.9%. In this study, all tumors were locally advanced (T3–4) regardless of the presence of perineural involvement, with a particularly higher incidence of T4 tumor involvement. They are independent poor prognostic factors for distant metastasis-free survival and locoregional relapse-free survival [24].

MRI has a sensitivity of 95–100% and a specificity of 85% in detecting PNS in head and neck cancers [25]. A recent meta-analysis showed FDG-PET/CT has a pooled sensitivity of 91.7%, pooled specificity of 92.35%, suggesting it is also an effective imaging modality [26]. MRI is more sensitive than CT and FDG-PET/CT in the detection of PNS due to better soft tissue contrast [27]. The excellent soft tissue contrast allows precise extent delineation for radiotherapy planning. However, the sensitivity for accurately depicting the entire disease extent is only about 63% compared to histology [21]. Pathological evidence shows that PNS is usually continuous, and imaging may not be able to detect the segment of nerves with low tumor burden [21]. There are also technical limitations from image acquisitions and image artifacts. From our experience, pre-contrast, non-fat-suppressed T1-weighted images in at least two planes and a volumetric T1-weighted post-contrast sequence with multiplanar reformatting play major roles in the imaging protocol to look for PNS. It is preferable to use a 3T magnet due to the ability to achieve a higher signal-to-noise ratio and thinner slices. Diligent search and scrutinization of the neural foramina and neural pathway for localizing the tumor spread are essential for accurate diagnosis and staging. Radiologists need to be skillful when scrutinizing skull base neuroforamina, as some of the foramina are better depicted on one plane than another [21]. Both anterograde and retrograde spread may occur, and the whole pathway needs to be evaluated as “skipped lesion” has been reported on imaging [21]. MRI signs for PNS include replacement of normal neural foramina fat by soft tissue signal, nerve enlargement, and nerve enhancement. Secondary imaging signs include denervation changes of involved musculature, with muscle edema in the acute phase and fatty atrophy in the chronic phase [23].

**Fig. 3:**
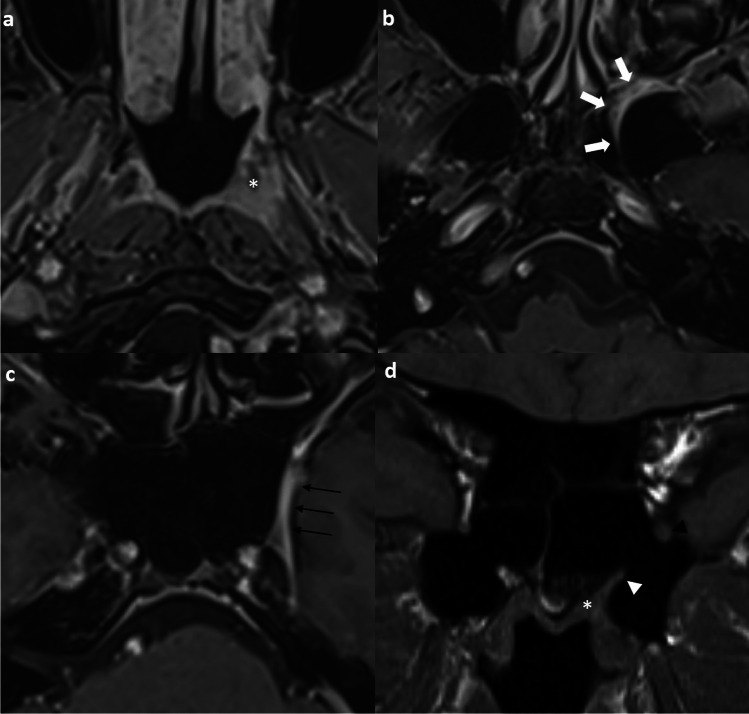
Perineural tumor spread in a 60-year-old man presented with left maxillary facial numbness with biopsy-proven undifferentiated carcinoma in left nasopharynx. Axial post-contrast T1-weighted image with fat suppression shows the left nasopharyngeal tumor at the fossa of Rosenmüller (asterisk) (**a**). Axial post-contrast T1-weighted image with fat suppression at a higher level reveals asymmetric enlargement and enhancement of the left vidian canal extending anterolaterally into the left pterygopalatine fossa (arrows) (**b**). Axial post-contrast T1-weighted MR image with fat suppression at a further higher level shows asymmetric enlargement and enhancement of the left foramen rotundum along the cavernous sinus (arrows) (**c**). Coronal T1-weighted image without fat suppression shows the asymmetric enlargement of the left foramen rotundum (black arrowhead) and vidian canal with fat effacement (white arrowhead). The nasopharyngeal tumor extends to the left vidian canal with perineural spread (asterisk) (**d**). The apparent small tumor in the nasopharynx with underdetection of perineural tumor spread may lead to understaging and subsequent undertreatment with missing irradiation target and over-estimation of survival

From the authors’ experience, not just locally aggressive tumors but also small localized tumors in the nasopharynx are seen to present with PNS on MRI. Accurate identification of PNS may upstage the disease to T4, allowing intensification of treatment with concurrent chemoradiation and exact conformation of the radiation field to the PNS pathway by IMRT to minimize residual disease and risk of local recurrence (Fig. 3).

4. *Nodal metastases of NPC follows a predictable and an orderly fashion. Nodal necrosis and radiological extranodal extension (ENE) carry significant prognostication and/ or treatment implications other than nodal staging itself.*

Nodal metastasis is a common presentation for NPC, occurring in about 85% of cases [8]. The nodal metastases follow an orderly pattern of spread, with the most commonly involved regions being the retropharyngeal (69%) and level II lymph nodes (70%) as echelon nodes. Despite the common perception of nodal spread from the retropharyngeal station to level II, isolated level II node involvement can occur without involvement of the retropharyngeal nodes, particularly for lateralized tumors in the nasopharynx [3, 8]. The level III (45%), IV (11%), and V (27%) nodal stations are less commonly involved [8]. Skip metastases are rarely observed, occurring in 0.5–7.9% of cases [8]. The presence of N2 or N3 disease, irrespective of any T stage according to the 8th American Joint Cancer Committee (AJCC) staging, should prompt the initiation of induction chemotherapy according to the latest National Comprehensive Cancer Network guidelines [28]. The presence of N3 disease also serves as an indication for FDG-PET/CT to evaluate for distant metastases [2].

Other low-risk nodal groups are rarely involved, such as the supraclavicular nodes (3%), level IB nodes (3%), level IA and VI nodes (0%), and parotid nodes (1%). This unusual nodal involvement should prompt a search for involvement of the relevant drainage area or possible differential diagnosis. For example, level IB involvement can occur with tumors involving the oral cavity, anterior nasal cavity, or when there is bulky disease in level II nodes (short axis > 20mm) leading to retrograde spread [7]. Parotid node involvement is uncommon in NPC unless there is an advanced-stage disease or again a bulky node in level II leading to retrograde spread [29]. Parotid node involvement is associated with a higher risk of treatment failure, distant metastasis, and regional recurrence, with similar disease-free survival (DFS) and distant metastasis-free survival (DMFS) as those with N3 disease [30]. The involvement of these nodal stations should be communicated to the radiation oncologists to ensure appropriate field coverage in radiotherapy planning [8, 29].

**Fig. 4:**
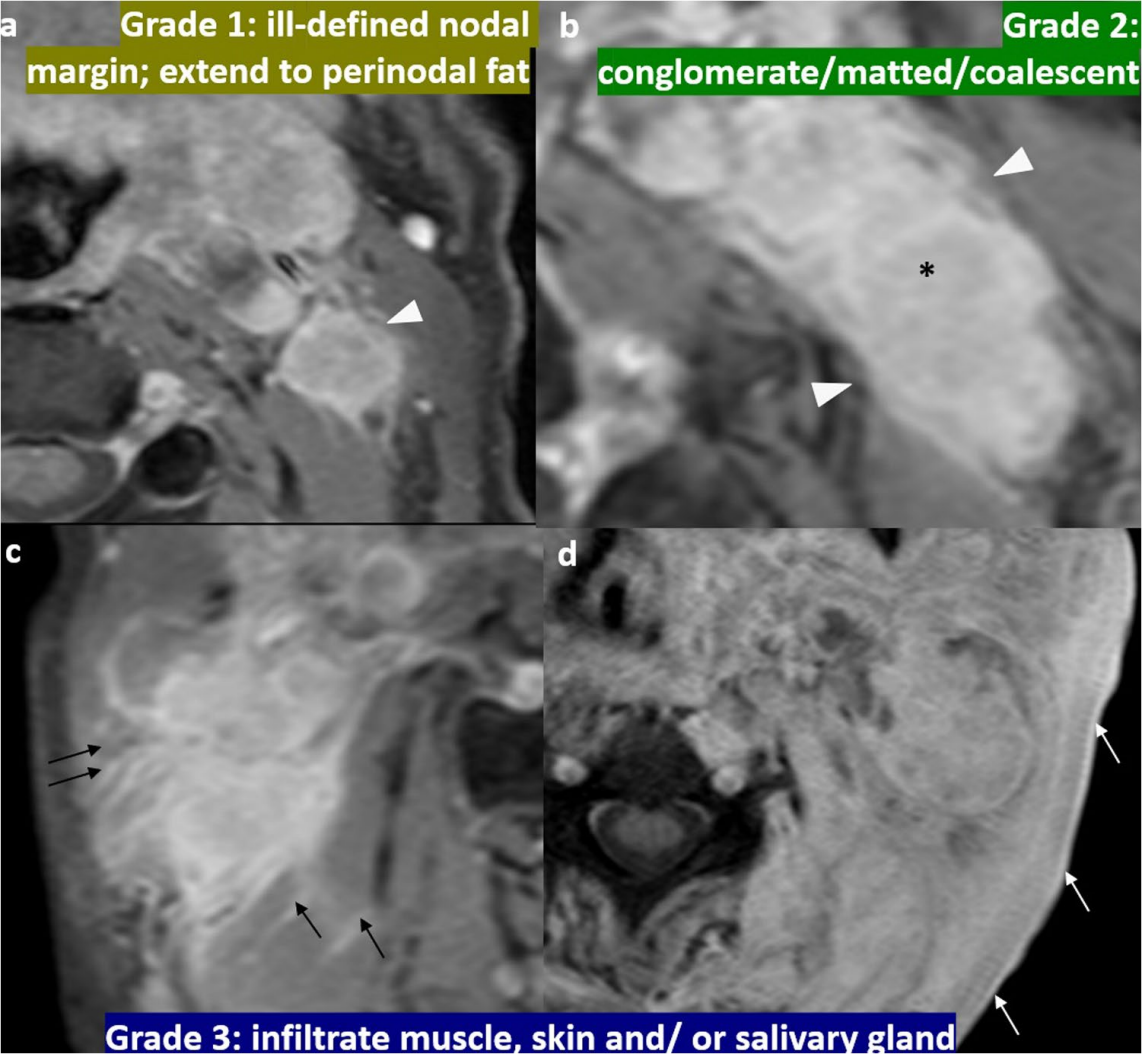
Grading of radiological extranodal extension from the Head and Neck Cancer International Group consensus recommendations [35]. Axial post-contrast T1-weighted image with fat suppression shows the left level II nodes demonstrating ill-defined nodal margin that extends into the perinodal fat (arrowhead). This is classified as grade 1 (**a**). Axial post-contrast T1-weighted image with fat suppression shows conglomerate left level II nodal mass (asterisk) with ill-defined nodal margin extending to the perinodal fat (arrowheads). This is classified as grade 2 (**b**). Axial post-contrast T1-weighted image with fat suppression shows conglomerate right level II nodal mass with extension to adjacent neck muscles (black arrows) (**c**). Axial post-contrast T1-weighted image with fat suppression shows conglomerate left level II nodal mass with extension to adjacent subcutaneous layer and skin (white arrows) (**d**). These are classified as grade 3

Other important features that could be identified on CT or MRI but not routinely included in the 8th AJCC staging system include cervical nodal necrosis and radiological ENE. They should be identified, reported, and communicated to oncologists for better patient risk stratification and prognostication. Cervical nodal necrosis has an incidence of 44% among NPC cases. It can be diagnosed as a hypoenhancing area within the node on CT, or as a focal area of high signal intensity on T2-weighted images or a focal area of low signal intensity on contrast-enhanced T1-weighted images, with or without a surrounding rim of enhancement [31]. It is an independent negative prognostic factor with a significantly poorer survival and a considerably higher distant metastasis rate [30]. A recent meta-analysis involving 4359 patients showed the presence of nodal necrosis predicted poorer DMFS, DFS, and overall survival (OS) [32]. ENE is a histological diagnosis referring to tumor growth beyond the capsule of a lymph node [33]. It reflects the aggressiveness of a tumor and has an incidence of 33.6%—39.8% in NPC [34]. In NPC, imaging serves as the primary modality for assessment of the presence of ENE, and radiological ENE is 90% specific to indicate the presence of pathological ENE [33]. It is important to recognize that radiological ENE appears as a spectrum of imaging abnormality, ranging from indistinct/ irregular nodal margin, extension to perinodal fat, conglomerate/ matted/ coalescent nodes and extension into adjacent structures such as muscle, skin and glands. Radiological ENE has an added value for risk stratification in future TNM but requires standardization in reporting [33, 35]. The recently published Head and Neck Cancer International Group consensus recommendations have established a grading system for reporting (Fig. 4) [35]. It is worth noting that infiltration of muscle, skin and glands (grade 3) is associated with a significantly shorter regional relapse-free survival, DMFS, and OS than those with ENE to fat or without ENE [34]. The addition of radiological ENE to N3 increases sensitivity to predict recurrence [36]. The advanced ENE (grade 3) has been proposed as a criterion of N3 in the 9th AJCC staging [37]. Reporting radiological ENE is important not only because it is an independent predictor of poor prognosis, it also requires the expertise of subspecialized head and neck oncologists in radiotherapy contouring. The presence of radiological ENE requires an expansion of 10mm CTV instead of 5mm around the GTV [8]. There should be prophylactic level 1B node coverage in low-risk CTV when a level II node with radiological ENE is present [8]. Muscle infiltration indicates the inclusion of the muscle near the node with at least a 10 mm margin in all directions [8].

5. *Various tumor mimics exist in the nasopharynx ranging from benign to malignant conditions. Accurate diagnosis avoids mismanagement.*

There are various tumor mimics in the nasopharynx mimicking NPC, ranging from benign conditions such as skull base osteomyelitis to malignant conditions such as nasopharyngeal lymphoma, invasive pituitary macroadenoma, and sphenoid sinus squamous cell carcinoma.

**Fig. 5:**
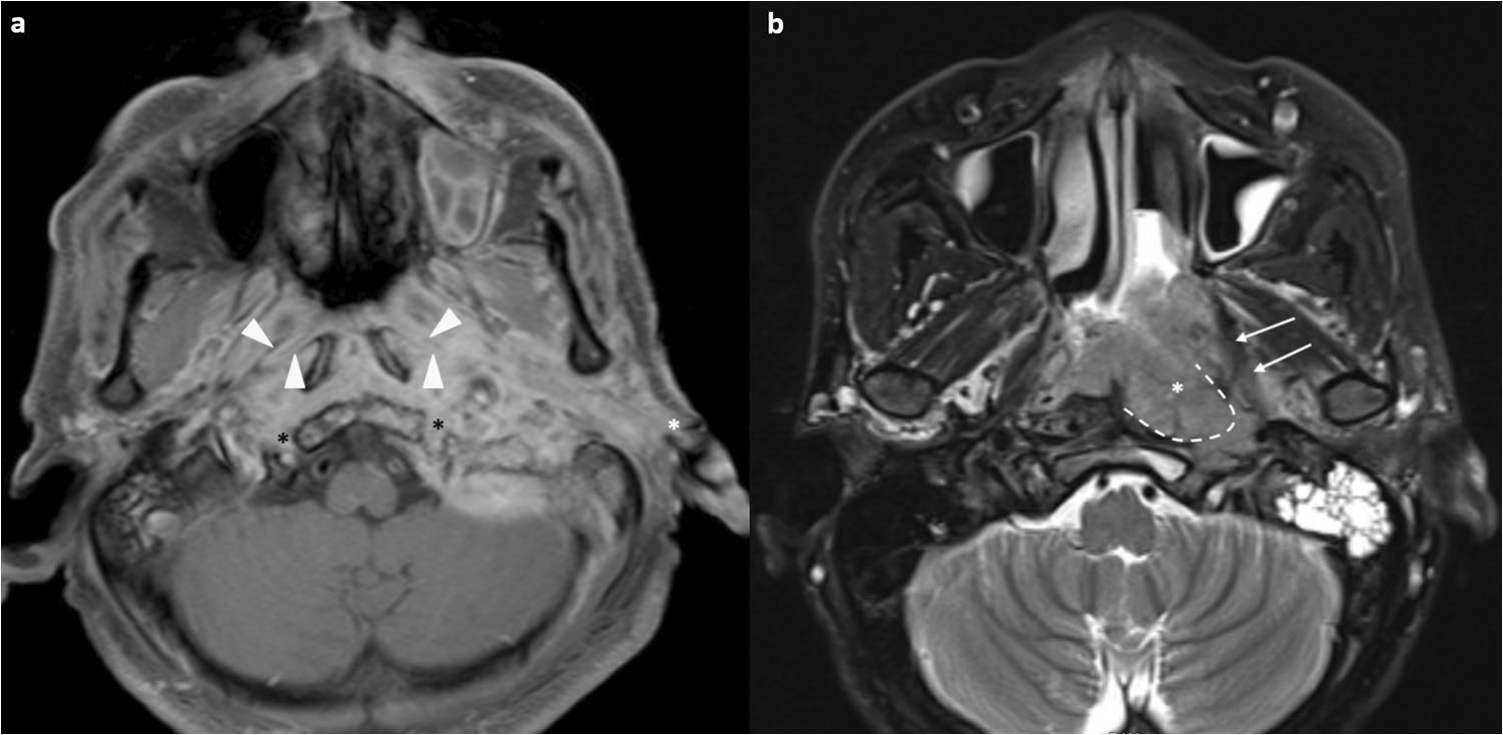
Illustrative comparative images of a 76-year-old female with skull base osteomyelitis (**a**) and a 53-year-old man with locally advanced NPC (clinical stage T4N3M0) (**b**). Axial post-contrast T1-weighted, fat-suppressed image shows intensely enhancing, infiltrative soft tissue signal involving the bilateral nasopharynx without significant mass effect. Note the preserved deep mucosal white line (arrowheads) and the preserved architecture of the nasopharynx. There is extension into the bilateral hypoglossal canals (black asterisks) and intracranial extension into the left posterior fossa. Lateral extension of the enhancing soft tissue signal to involve the left parotid gland is observed (white asterisk). Extensive enhancing marrow signal is noted in the bilateral basioccipital, left petrous and tympano-mastoid temporal bone. No retropharyngeal or cervical lymphadenopathy (**a**). Axial T2-weighted, fat-suppressed image shows the characteristic intermediate signal for NPC (asterisk). There is associated mass effect especially on the left nasopharynx and left prevertebral muscle. Disruption of the normal architecture of nasopharynx is evidenced by the partial disruption of the pharyngobasilar fascia (dotted line). Limited left lateral extension of the tumor is observed as the tumor is still bound by the tensor veli palatini muscle (arrows) (**b**). Extensive bilateral cervical lymphadenopathies (not shown)

Skull base osteomyelitis (SBO) is a rare and life-threatening complication of otogenic infection in elderly patients with diabetes mellitus [38–40]. It is usually a sequela of untreated necrotizing external otitis caused by *Pseudomonas aeruginosa* [38, 39]. It poses a diagnostic challenge to clinicians and radiologists and a delayed diagnosis of up to 6 months is common [38, 39]. MRI is often the investigation of choice for establishing the diagnosis and depicting the disease extent. NPC is the main differential diagnosis to consider [39, 41] and several helpful distinguishing imaging signs are illustrated (Fig. 5) (Table 2). Endoscopic biopsy or intraoperative image-guided biopsy is required in equivocal cases [38, 41].

**Table 2:** Distinguishing MR features between skull base osteomyelitis and NPC

Skull base osteomyelitis	NPC
Infiltrative process without destruction of fascial plane Preserved pharyngobasilar fascia and deep white mucosal line	Displaces or replaces normal anatomy without preservation of tissue planes Disruption of pharyngobasilar fascia and deep white mucosal line
Lateral extension to the deep lobe of left parotid and bilateral pterygoid muscles	Spreads in an orderly fashion through pathways of lesser resistance
Intracranial spread and preferentially involves lower cranial nerves foramina	Skull base extension ± intracranial spread; Foramen lacerum and ovale are more preferentially affected than lower cranial nerve foramina
Heterogenous T2W signal with high signal areas representing edema; Abnormal bone marrow signal and soft tissue enhancement	Characteristic intermediate T2W signal
Higher ADC value	Restricted diffusion with lower ADC value
No retropharyngeal or cervical lymphadenopathy	Retropharyngeal and cervical lymphadenopathy
Mastoid effusion with soft tissue thickening at external auditory canal (EAC)	May see mastoid effusion but typically no EAC involvement
Abscess formation	No abscess

**Fig. 6:**
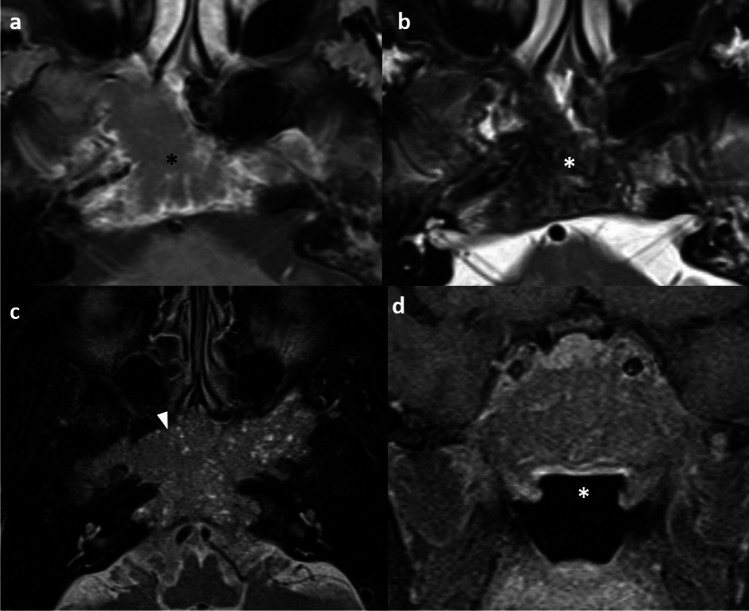
Sphenoid sinus squamous cell carcinoma in a 63-year-old man presented with headache and right 6th nerve palsy (a & b). Initial plain CT brain showed a hyperdense right sphenoid mass with clival destruction (not shown). Axial post-contrast T1-weighted image with fat-suppression shows an irregular and infiltrative mass centered at right sphenoid sinus with clival, bilateral petrous apex destruction, and intracranial extension (not shown). The lesion shows irregular peripheral enhancement with central areas of hypo-enhancement (asterisk) (**a**). Axial T2-weighted image with fat-suppression shows the corresponding mass with heterogenous, predominantly hypointense signal (asterisk) (**b**). Invasive pituitary macroadenoma in a 73-year-old woman presented with persistent headache (c & d). Axial T2-weighted image with fat-suppression shows an extensive tumor mass with intermediate signal containing multiple internal cystic foci involving the central skull base (arrowhead) (**c**). Coronal post-contrast T1-weighted image with fat-suppression shows the lesion centered at the sella turcica and clivus with bilateral cavernous sinus extension. The nasopharyngeal roof is unremarkable (asterisk) (**d**)

Nasopharyngeal lymphoma and NPC differ in their imaging morphology. Lymphoma tends to appear as a midline mass with symmetry and involvement of Waldeyer’s ring while NPC tends to be an asymmetric mass of characteristic signals [42]. Lymphoma presents as an exophytic mass filling the airway with rare skull base invasion while NPC shows propensity for skull base invasion with or without intracranial spread [42]. The presence of parotid lymphadenopathy is more commonly observed in lymphoma than NPC [42]. Lymphoma tends to show significantly lower ADC values than NPC [42, 43]. Invasive pituitary macroadenoma, and sphenoid sinus squamous cell carcinoma tend to show different epicenters and signal characteristics as NPC (Fig. 6).

## Post-treatment imaging

- *#1 Recurrent tumors most commonly are in-field failure and a distinction has to be made with various post-treatment changes. Additional challenges in imaging interpretation are related to delayed tumor regression and time lag between histological and radiological regression. Identification of post-treatment changes from residual or recurrent tumors can avoid unnecessary biopsies.*

Despite the advancement of IMRT techniques for the primary treatment of NPC, about 5–10% of patients will experience local or regional recurrence after treatment [44, 45]. Direct visualization by endoscopy is challenging and may miss 27.8% of tumors due to submucosal or deep-seated locations [46]. Besides, endoscopic biopsy may have a risk of sampling errors when residual tumors are present only in small clusters [47]. Consequently, post-treatment imaging surveillance is a vital component of overall evaluation. The primary responsibility of radiologists in this phase is to identify the presence of any recurrent tumors. This is challenging given the complexity of various post-treatment anatomical distortions and changes. Knowledge of the characteristic signals of recurrent nasopharyngeal tumors and their failure patterns in the era of IMRT guides effective image interpretation. Key considerations to be addressed include selecting the appropriate imaging modality, determining the optimal timing for imaging, knowing what to expect on the post-treatment images, and finally deciding on the course of action when a recurrent tumor is detected.

The latency period for recurrent NPC is wide, with a median latency of 1.9 years (range 0.6–11.9) [48]. Most recurrences (81.7%) occur in the first three years after IMRT [45]. However, there is still a small portion of recurrence that occurs more than 5 years post-treatment, and therefore continual surveillance is important. In-field recurrence (93.3%) is the most common pattern for loco-regional failure of NPC treatment [45]. Distant metastases also occur in a certain portion of patients (17.5% of the cases) and remain the major cause of mortality [46].

Initial components for evaluation include clinical assessment, endoscopy, and plasma EBV DNA levels. Both MRI or FDG-PET/CT can be used for imaging response assessment. MRI is the modality of choice for local surveillance given its superior soft tissue contrast for assessment of local anatomical distortion, as well as disease extent in case of recurrent tumors. Accurate restaging and mapping of tumor extent forms an essential foundation for salvage therapy planning [48].

**Fig. 7:**
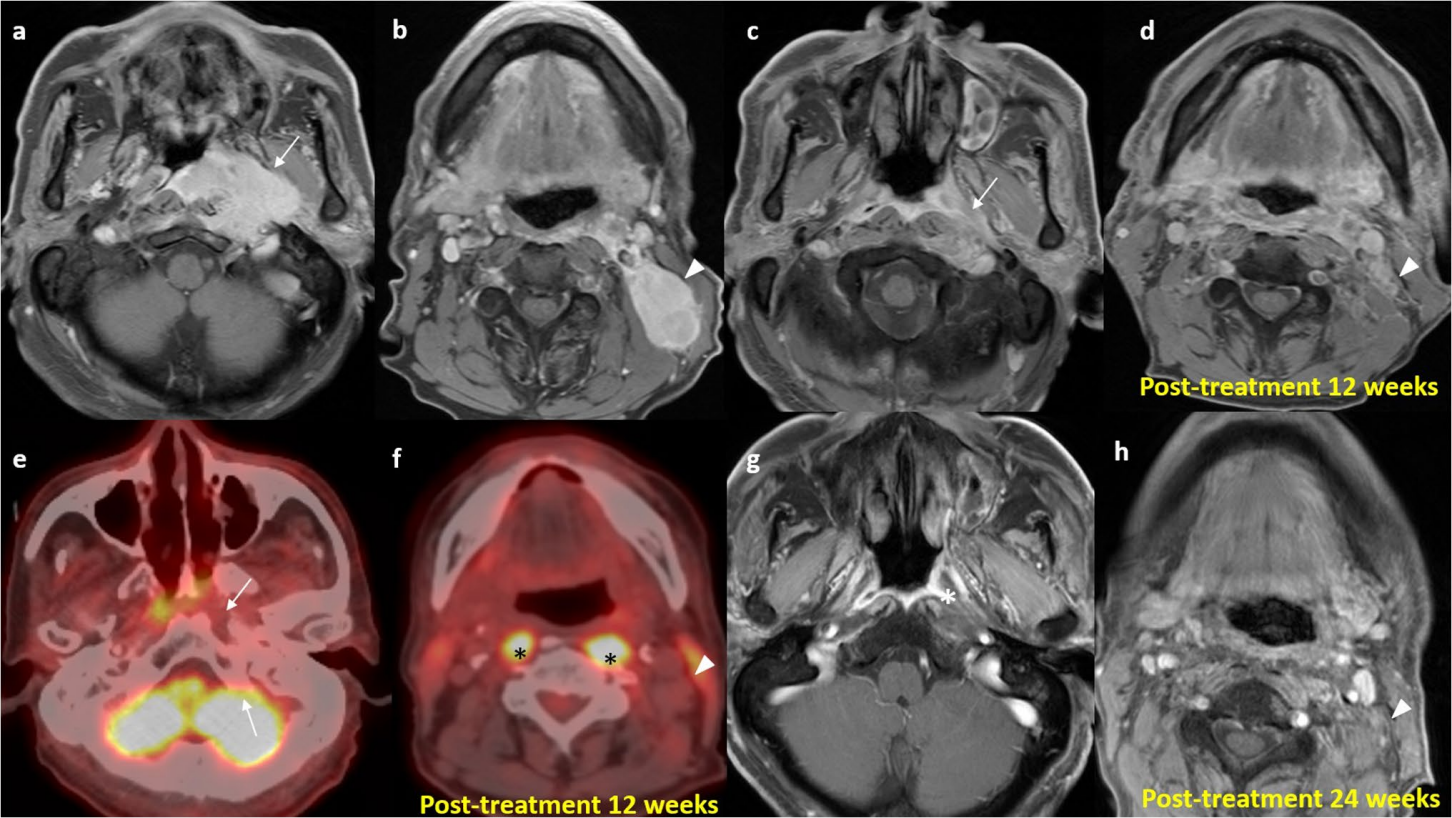
Delayed tumor regression and time lag between anatomical and metabolic regression in a 69-year-old man with locally extensive NPC (clinical stage T4N3M0) completed chemo-irradiation in 2023. Axial T1 post-contrast images with fat-suppression at pre-treatment stage show a large and infiltrative enhancing left nasopharyngeal tumor (arrow) and left level II lymphadenopathy (arrowhead) (**a**) & (**b**). There was evidence of pathological complete remission in the nasopharyngeal tumor at 10 weeks after completion of treatment. Axial T1 post-contrast images with fat suppression at 12 weeks show interval shrinkage in size of the known tumor with residual enhancing soft tissue in the left retropharyngeal and prevertebral space (arrow). The left level II node also shows interval shrinkage with residual heterogeneously enhancing soft tissue (arrowhead) (**c**) and (**d**). These were equivocal for residual tumors, which might warrant further palliative treatment. Axial fusion FDG PET/CT images at 12 weeks show the left nasopharyngeal lesion (arrow) as well as the left level II lesion (arrowhead) to be non-FDG avid, suggestive of metabolic resolution, despite the small residual soft tissue density. Apparent muscle activity along the bilateral longus colli muscles (asterisks) is a known diagnostic pitfall mimicking disease recurrence (**e**) and (**f**). FDG PET/ CT can be a helpful tool in this occasion due to its high negative predictive value. The patient subsequently underwent clinical observation instead of unnecessary biopsy or oncological therapy. Axial T1 post-contrast images at 24 weeks show almost complete resolution of the left nasopharyngeal lesion (asterisk) and the left level II node (arrowhead) (**g**) and (**h**)

The optimal timing of follow-up imaging is a debated topic. International guidelines recommend that baseline follow-up imaging be done 3 to 12 months upon treatment completion [28, 49]. The basis of scheduling post-treatment imaging originates from a study in 1999 showing that 93.2% of NPC patients treated with three-dimensional conformal radiotherapy can achieve histological remission by 12 weeks post-treatment [50]. Another study in 2017 performed for post-treatment imaging after IMRT shows that only 83.3% of patients achieves complete clinical response by MRI and flexible endoscopy in 3–4 months, while this figure increases to 91.4% at 6–9 months. The prognosis of these two groups of patients showed no significant difference, therefore it is suggested that 6–9 months may be the optimal time point for maximal tumor response after IMRT [51]. Thus, delayed primary tumor regression and a time lag between histological and radiological regression can be potential pitfalls in follow-up imaging [47]. Anatomical regression also lags behind metabolic regression. Therefore, residual treated tissue is expected in the early phases of imaging surveillance and does not necessarily indicate the presence of viable tumor cells [46, 52]. In a longitudinal cohort of 19 patients with "phantom tumor phenomenon" on MRI, 73.7% of them had histological correlation showing necrosis, inflammation, or reactive epithelial cells [53]. These false positive mimicking residual or recurrent tumors on MRI result in unnecessary biopsies, or even re-irradiation and surgery [53]. The optimal timing of the post-treatment MRI is a balance between early detection of residual or recurrent disease versus reduction of indeterminate rates in radiology reports. Most patients will require more than one post-treatment MRI to achieve a definitive disease status [47]. Only 33.2% cases will show complete remission on the first post-treatment MRI performed at a median time of 93 days (range 32–346 days), with the remaining cases either indeterminate (50.2%) or showing partial response (16.6%), requiring a second post-treatment MRI for follow-up [47] (Fig. 7). Scheduling the first post-treatment MRI from 3 months to around 4 months upon treatment significantly increases the proportion of patients with radiological complete remission (32.8% vs 83.3%) [47].

**Fig. 8:**
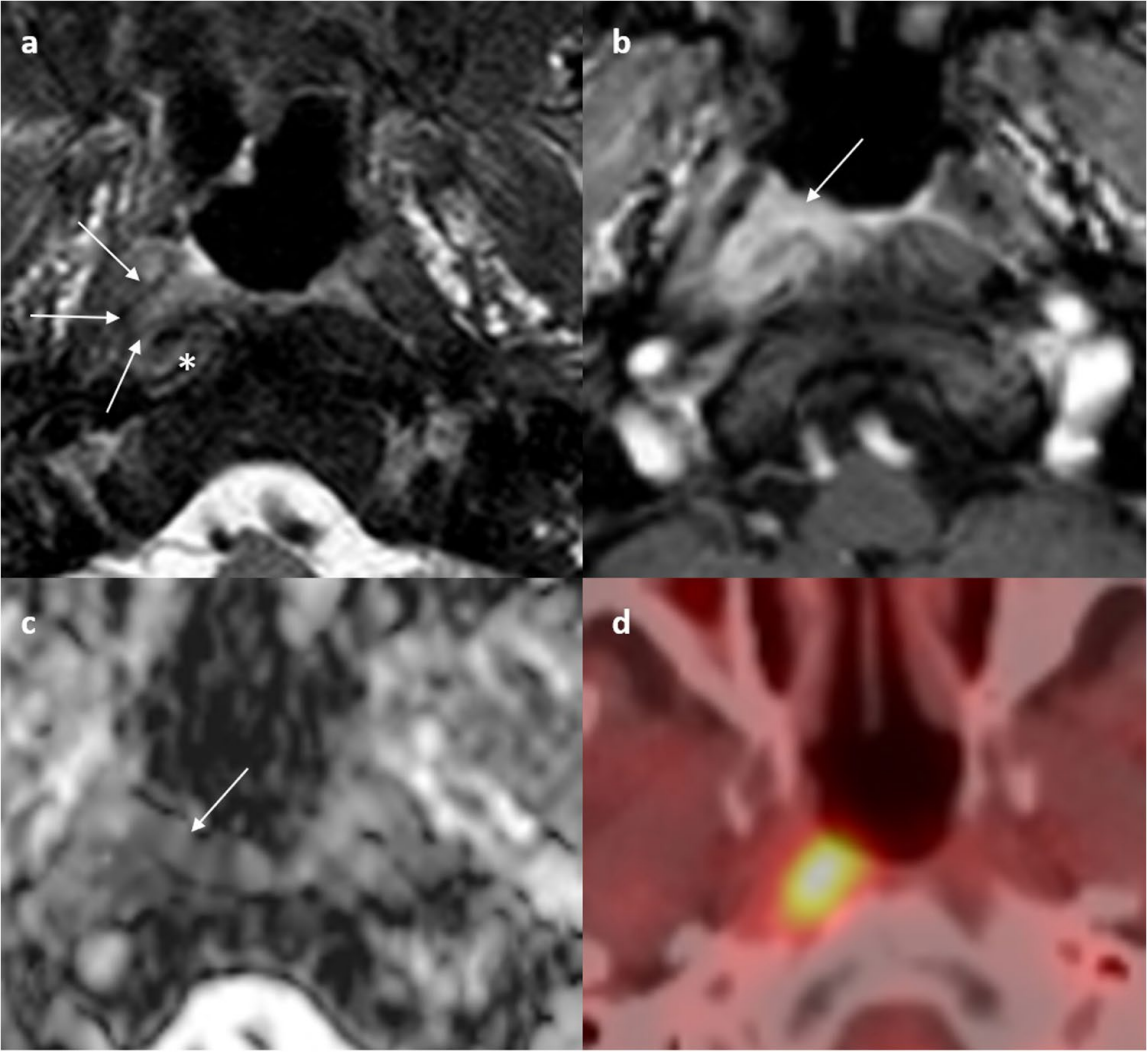
Typical signal characteristics of the recurrent nasopharyngeal tumor in a 72-year-old man with history of NPC (clinical stage T1N1M0) and radical radiotherapy completed in 2013. Axial T2-weighted image with fat-suppression shows an intermediate signal mass in the right nasopharynx causing focal expansion of the pharyngobasilar fascia which appears ill-defined (arrows). There is also extension of signal abnormality to the right prevertebral muscle (asterisk) (**a**). Axial T1-weighted image with fat-suppression shows the moderately enhancing mass in the right nasopharynx (arrow) with extension to right prevertebral muscle (arrow) (**b**). On the ADC map there is hypointense signal corresponding to the right nasopharyngeal mass (arrow) (**c**). Axial fusion FDG-PET/CT confirms the presence of a hypermetabolic mass in the right nasopharynx (**d**). Biopsy confirmed recurrent NPC

Radiologists must be aware of these pitfalls when interpreting the MRI. Conventional MRI is limited in the differentiation of the irradiated nasopharynx with anatomical distortion, granulation tissue, necrosis, and true viable tumor [54]. Local anatomical distortion includes loss of symmetry of the nasopharynx and partial effacement of the lateral pharyngeal recess, posing challenges in assessment. Besides, a residual mass on follow-up MRI may not contain a viable tumor [54]. Non-enhancing tissue with a dark signal on T2-weighted imaging represents mature fibrosis. It may be difficult to distinguish between immature fibrosis, granulation tissue, and residual or recurrent tumors, as all of them enhance [55]. Correlation with endoscopic findings is important in imaging interpretation. Recurrent tumors typically show intermediate signal intensity on T2-weighted imaging with moderate contrast enhancement on T1-weighted contrast-enhanced images without fat saturation, and restricted diffusion on DWI [48] (Fig. 8).

**Fig. 9:**
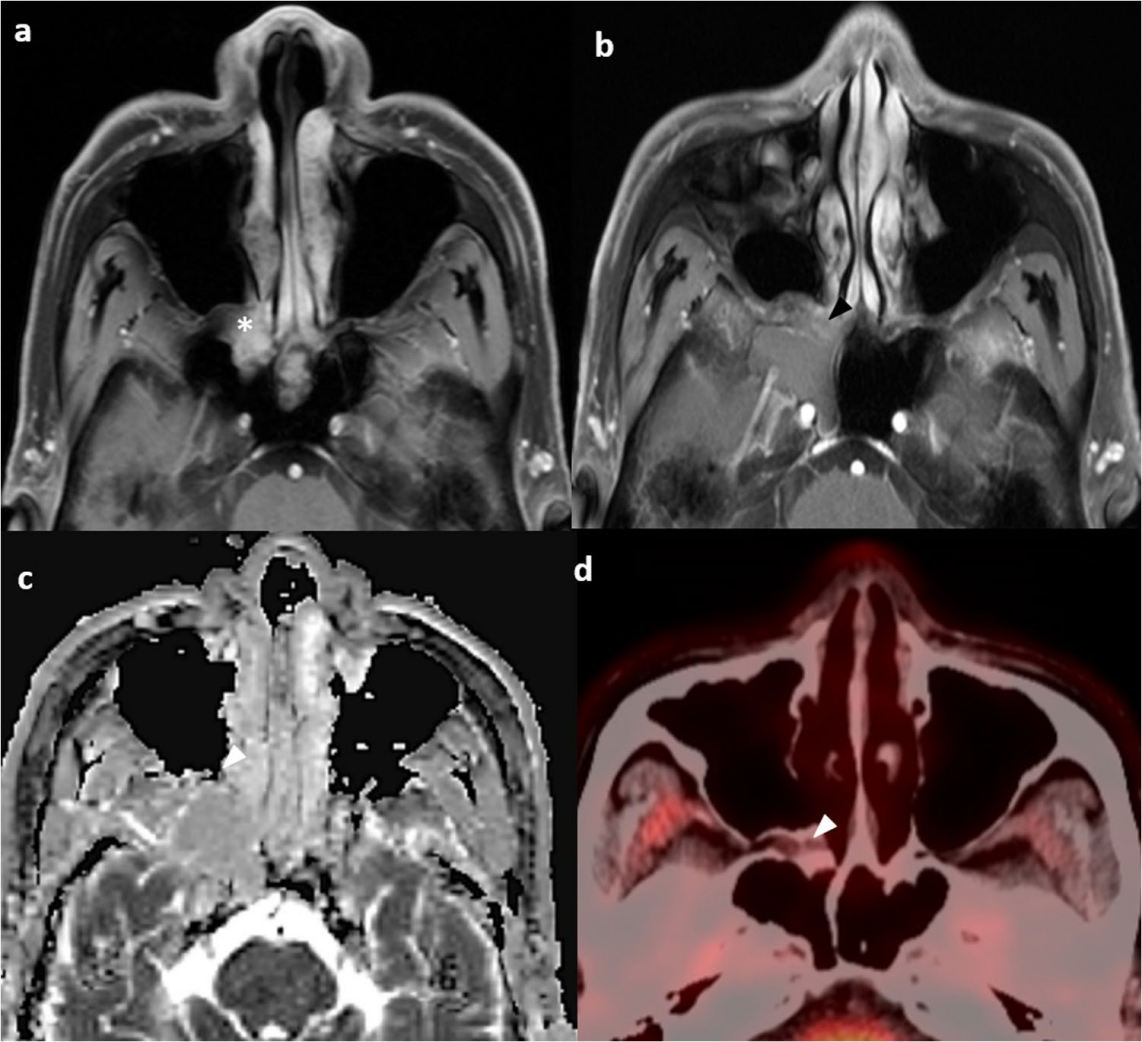
Scar tissue in a 65-year-old man who had NPC (clinical stage T3N1M0) involving the right pterygopalatine fossa completed combined chemoirradiation in 2022. Post-treatment endoscopy revealed persistent tumor and therefore stereotactic radiotherapy was given. Axial T1-weighted post-contrast image with fat suppression shows the enhancing soft tissue at the right pterygopalatine fossa with local expansion (asterisk) suggestive of tumor (**a**). Axial T1-weighted post-contrast image with fat suppression performed at 18 months upon treatment completion shows interval shrinkage of the previous noted tumor at right pterygopalatine fossa but with persistent mildly enhancing soft tissue thickening (arrowhead) equivocal for residual tumor or scar (**b**). ADC map of the diffusion-weighted imaging performed at 18 months upon treatment completion showed no restricted diffusion in the right pterygopalatine fossa (arrowhead) (**c**). Axial fusion FDG-PET/CT at 18 months post-treatment depicts the right pterygopalatine fossa soft tissue to be non-FDG avid (**d**). The original tumor at the right nasopharynx has resolved (not shown). Findings are suggestive of scar tissue without viable tumor

A practical solution includes the use of DWI and/ or FDG-PET/CT (Fig. 9). DWI is helpful in addition to conventional MRI for the differentiation between benign post-treatment change and fibrosis versus tumor, as the latter will show restricted diffusion [56, 57]. Quantitative measurement of ADC value has been proven useful in the differentiation of benign post-treatment changes and fibrosis versus residual or recurrent tumors. The use of an ADC cutoff of 887 × 10^–6^ mm^2^/s has a sensitivity of 87.2%, specificity of 94.1% and an area under curve of 0.967 in this regard [57]. FDG-PET/CT has a very high negative predictive value of 95% for recurrent tumors, and a negative PET-CT at 6 months following completion of treatment indicates that residual or recurrent disease is highly unlikely [56]. Thus, it can be particularly useful in cases of indeterminate findings related to delayed tumor regression.

These limitations also support the fact that the combined use of both MRI and FDG-PET/CT is significantly more accurate than the use of either modality individually in detecting residual or recurrent NPC [58]. PET/MRI may be a useful one-stop investigation with both complementary anatomical and functional information, but there are technical challenges to overcome in combining PET and MRI such as MRI-based attenuation correction, clinical workflow integration, and motion artifacts owing to the long scanning time [59].

Identification of recurrent tumors should be followed by careful scrutiny to map out the full extent of the tumor and its nodal metastases, if any, for restaging. Attention should be paid to the relationship of the recurrent tumor and the internal carotid artery, as it determines the operability and the approach of salvage surgery [48, 60]. A close resection margin may limit the option of endoscopic salvage [60]. Nodal metastases should be evaluated by both MRI and FDG-PET/CT. Ultrasound-guided fine-needle aspiration is helpful for equivocal nodes. Selective neck dissection is the preferred treatment option due to better functional outcome [61], therefore, accurate detection of nodal metastases is crucial. Systemic staging for distant metastases by FDG-PET/CT is vital, as the treatment strategy shifts from radical surgery to palliative chemotherapy if distant metastasis is present.

- *#2 Post-radiation nasopharyngeal necrosis is a rare but life-threatening delayed complication mimicking tumor recurrence to be recognized.*

Post-radiation nasopharyngeal necrosis is a rare, delayed but life-threatening complication following radiation therapy for nasopharyngeal tumors [62]. It is a form of soft tissue necrosis involving the surrounding and affiliated tissues of the nasopharynx, such as the mucosa, longus capitis muscles, parapharyngeal tissues, and skull base [62]. The incidence is about 1–2% in patients with NPC receiving primary radiotherapy, but increases to 30–40% after re-irradiation [63]. Predominant symptoms include foul nasal odor, persistent headache, and nasal hemorrhage [64]. Erosion of ICA may lead to potentially fatal hemorrhage [64].

A three-stage system for correlation of clinical and endoscopic findings has been described in the literature [62, 64]. The first stage mainly involves primary endoscopic findings of local mucosal structural alteration including ulceration. The second stage involves surrounding necrotic soft tissue on endoscopy, and the most severe stage involves osteonecrosis, with patients presenting with persistent headaches and foul odor [64].

Although endoscopic and imaging findings may be characteristic, the diagnosis remains challenging. Histopathological examination remains the gold standard to support the diagnosis and exclude the presence of recurrent tumors. Radiation-induced fibrosis is the main mechanism leading to the development of post-irradiation nasopharyngeal necrosis [63]. Distinctive radiation-induced or radiation-associated stromal changes are best recognized by the presence of fibrosis, fibrinous exudate, atypical fibroblasts, and paucity of cellular inflammatory exudate, which are characteristic findings [65].

**Fig. 10:**
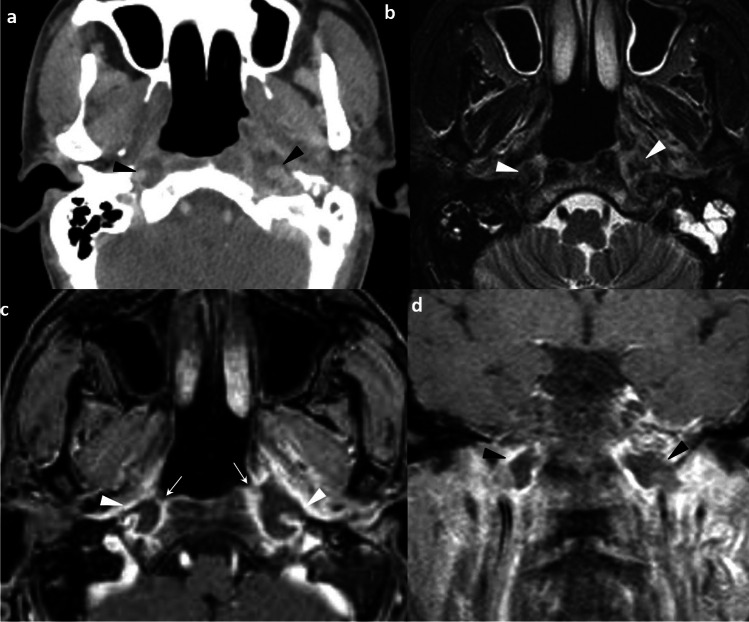
Post-radiation nasopharyngeal necrosis in a 49-year-old man with NPC (clinical stage T4N2M0). He developed epistaxis and progressive headache 6 months after radiotherapy. Nasoendoscopy showed crusted and ulcerated mucosa without mass. Axial contrast-enhanced CT image shows mucosal irregularity in bilateral nasopharynx. Rim-enhancing lesions are seen in bilateral nasopharynx with extension to carotid spaces encasing the internal carotid arteries (arrowheads) (**a**). Axial T2-weighted image with fat suppression shows the lesions to be heterogeneous in signal intensity (arrowheads) (**b**). Axial and coronal post-contrast T1-weighted image with fat suppression show focal defects in the bilateral nasopharynx as discontinuity of the deep mucosal line (arrows). The rim-enhancing lesions encasing the bilateral internal carotid arteries are again seen (arrowheads) (**c**) and (**d**). Deep biopsy was suspended due to the close relationship with internal carotid arteries. The lesions gradually resolved on conservative management with restoration of the continuity of the deep mucosal white line on follow-up MRI (not shown)

Characteristic MRI features include local defects in the nasopharyngeal wall and rim-enhancing areas with central non-enhancement [62, 64, 65] (Fig. 10). It is important to scrutinize the internal carotid artery to look for pseudoaneurysm formation. CT may also depict gas densities presumed to be have tracked from the ulceration to the submucosal soft tissue. DWI may show restricted diffusion and FDG-PET/CT may show hypermetabolism [65]. These features overlap with those of recurrent tumors, potentially leading to misdiagnosis [65]. However, it is uncommon for recurrent nasopharyngeal tumors to show internal hypoenhancing areas related to necrosis. Understanding of this rare entity is important for early recognition and diagnosis. Imaging may also guide potential site for biopsy, which carries a higher risk if there is deep ulceration or if the internal carotid artery is exposed.

No specific treatment has been established other than conservative therapy, including nasal irrigation, systemic or topical antibiotics, intravenous nutritional supplements, hyperbaric oxygen, and debridement guided by nasal endoscopy [63]. Endoscopic or open surgery has also been suggested to remove necrotic tissue, followed by flap coverage; however these approaches are limited by available expertise [63]. The prognosis remains poor, with a reported 2-year overall survival of 51.6% [63].

- *#3 Not every enlarging retropharyngeal mass is a sign of malignancy: the hypertrophied superior cervical ganglia.*

The superior cervical ganglion (SCG) can be mistaken for an enlarged retropharyngeal lymph node in patients with post-treatment NPC, leading to unnecessary surgery [66]. Recent case reports and studies have shown that the SCG has a propensity to become hypertrophic after radiotherapy, leading to their misidentification on imaging as metastatic lateral retropharyngeal lymph nodes [67, 68].

Several key MRI features help in the discrimination. SCGs are usually medial to the internal carotid arteries and lateral to the prevertebral muscles, between the C2 and C4 vertebrae [68]. They typically exhibit a fusiform or elongated shape on the coronal plane, with a characteristic and consistent (> 90%) central dot sign best appreciated on T2-weighted and post-contrast T1-weighted images with fat-suppression, related to the underlying venule [66, 68, 69]. SCGs show homogeneous and intense enhancement [69]. After radiotherapy, the size, T2-weighted signal, and the ADC value can increase up to about 1 year and then remain stable [69, 70]. The intraganglionic hypointensity, homogeneous enhancement, and well-defined margin should be maintained [69]. The enlargement is thought to be related to Schwann cell proliferation and/ or thickening of the perineurium and epineurium in response to radiation-induced damage [70].

**Fig. 11:**
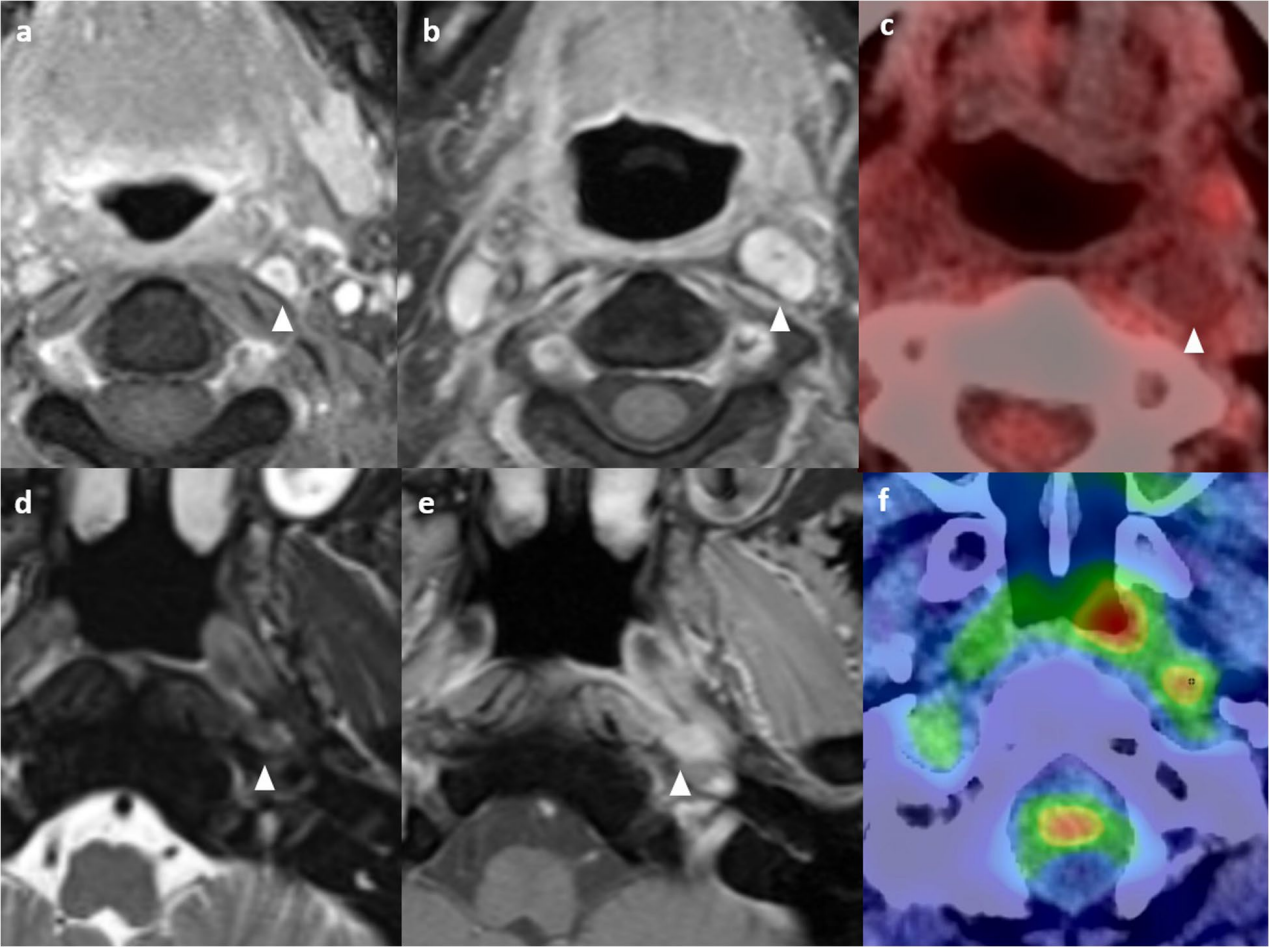
Illustrative comparative images of a 49-year-old female with a hypertrophied superior cervical ganglion with history of treated NPC in 2014 (clinical stage T4N1M0) (**a**) to (**c**) and a 58-year-old man with recurrent retropharyngeal node 2 years post-treatment of locally advanced NPC (clinical stage T4N3M0) (**d**) to (**f**). Axial T1-weighted post-contrast image with fat suppression at 6 months post-treatment shows a sub-centimeter enhancing lesion with central hypointense dot at left retropharyngeal space (arrowhead) located anteromedial to the ipsilateral internal carotid artery (**a**). The lesion showed interval enlargement and then stabilized at 6 and 7 years upon treatment completion (not shown). Axial post-contrast T1-weighted image with fat suppression at 9 years post-treatment shows an enlarged lesion in the left retropharyngeal space (arrowhead). Its T2-weighted hyperintense signal persists. The internal architecture with central hypointense dot is maintained (**b**). Otherwise, the nasopharynx showed no mass all along to indicate recurrence (not shown). Axial FDG-PET fusion image shows the lesion being non FDG-avid (**c**). Overall findings are in keeping with a hypertrophied superior cervical ganglion after radiotherapy. Axial T2-weighted image with fat-suppression at 2 years post-treatment shows a new sub-centimeter left retropharyngeal node with intermediate signal intensity (arrowhead) (**d**). Axial T1-weighted post-contrast image with fat suppression at 2 years post-treatment shows the corresponding lesion to be avidly enhancing (arrowhead) (**e**). Axial FDG-PET fusion image shows the lesion being hypermetabolic (**f**). No other local recurrence or distant metastases was depicted. The patient underwent salvage open nasopharyngectomy and confirmed recurrent tumor in the retropharyngeal node

To distinguish SCG from a retropharyngeal lymph node, the location, size, and signal characteristics on MRI are important considerations [71]. SCG is typically located more posterolaterally and inferiorly than a retropharyngeal node, which is most often (about 75%) located at the level of C1 [8]. SCG tends to be larger in volume, has a higher degree of contrast enhancement, and a significantly higher ADC value than a retropharyngeal node (1.80 ± 0.28 × 10^−3^ mm^2^/s versus 0.73 ± 0.10 × 10^−3^ mm^2^/s, *P* < 0.001) [65]. FDG-PET/CT can be another problem-solving tool, as SCG usually (> 80%) does not show FDG avidity. (Fig. 11).

- *#4 Skull base marrow signal change after radiotherapy does not always equal recurrent or residual tumor.*

**Fig. 12:**
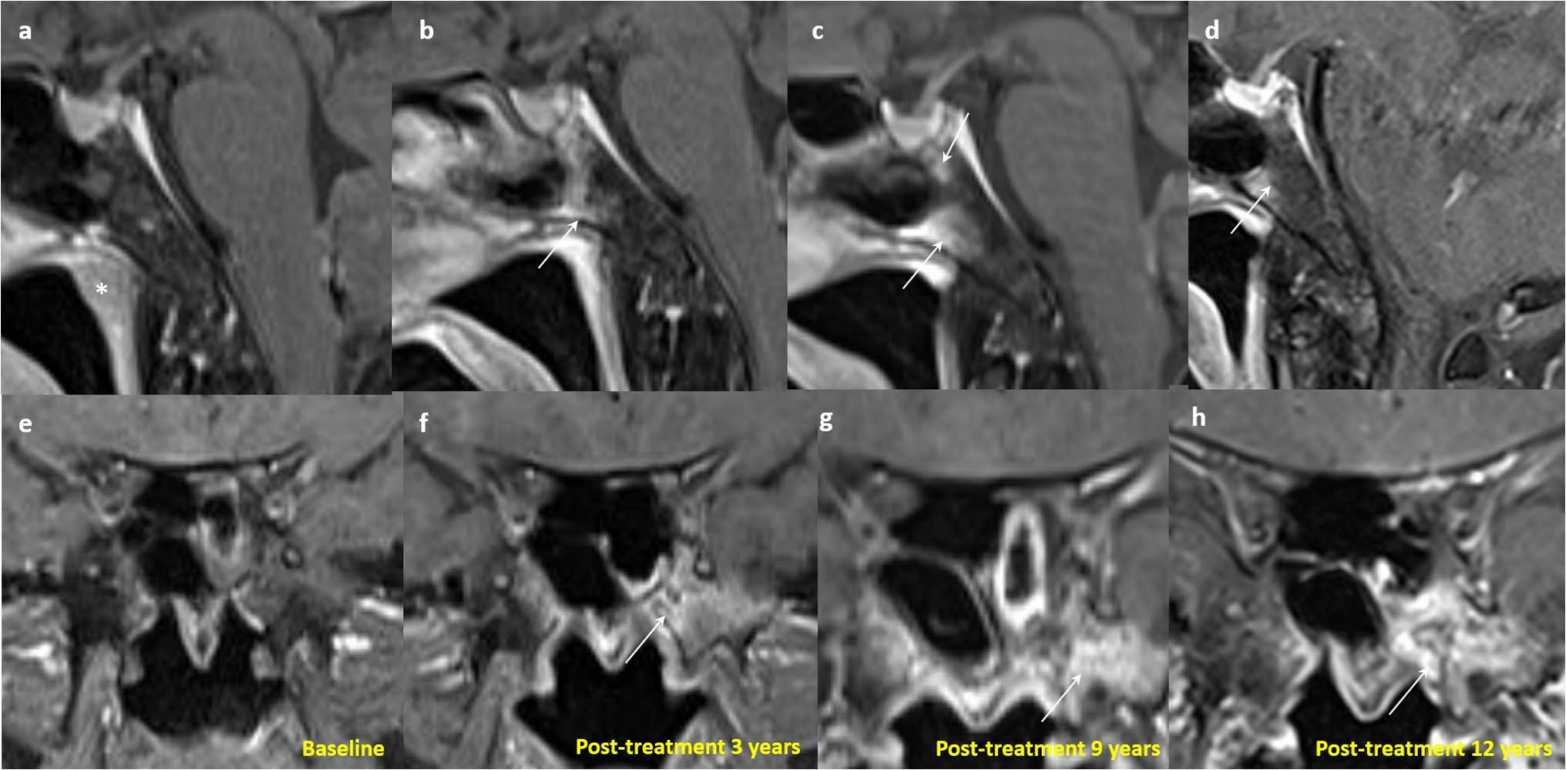
Serial MRI demonstrating the signal change in the basisphenoid of a 56-year-old female with a history of localized NPC (clinical stage T1N0M0) in 2011 with pathological complete remission. Sagittal T1-weighted post-contrast MRI with fat suppression at baseline shows no evidence of signal abnormality at the clivus. The nasopharyngeal tumor is seen (asterisk) (**a**). There is progressive increase in the patchy enhancement in the clivus in 3-year and 9-year follow–up images (**b**) and (**c**), and the signal abnormality shows partial resolution in 12-year follow-up image (arrows) (**d**). Coronal T1 post-contrast MRI with fat suppression at baseline shows no evidence of signal abnormality at the basisphenoid (**e**). There is progressive increase in the patchy enhancement in the bilateral basisphenoid, more on left side, in 3-year follow-up image (**f**) and 9-year follow–up image (**g**), and the signal abnormality shows partial resolution in 12-year follow-up image (arrows) (**h**)

In post-treatment MRI, surveillance of skull base marrow signal is important as it may be one of the first signs of tumor recurrence. Evaluation is challenging not only because of the slow recovery of the previously infiltrative bone marrow, but also due to the appearance of new bone marrow lesions related to radiation-induced injury (e.g. radiation osteitis, osteoradionecrosis (ORN)), or signal changes related to granulation tissues and fibrosis [72]. In fact, skull base and clival marrow signal change can appear on post-treatment MRI with or without preceding tumoral invasion, and this can persist for many years after treatment completion [72] (Fig. 12). Moreover, these signal changes are also nonspecific for residual/recurrent tumor or benign post-treatment changes.

A retrospective study reviewing the serial MRIs of 50 patients with NPC showed that clival bone marrow signal change persisted without any evidence of recurrence in 26 patients with clival infiltration at diagnosis, for a mean of 66.5 (maximum 137) months. There was accompanying contrast enhancement noted for up to 125 months [72]. The presence of enhancing marrow signal in the post-irradiated skull base does not always indicate recurrent or residual tumor, and it is most often benign post-treatment changes.

There are several approaches to this diagnostic dilemma. Radiation osteitis is often asymptomatic and incidentally detected [73]. It appears as foci of low, intermediate and high signal intensity on T2-weighted images, with patchy areas of enhancement [73]. Patients with recurrent NPC may present with bloody nasal discharge, headache or painless enlargement of a neck node [74], and may be accompanied by a rising trend of plasma EBV DNA [75]. The time frame of occurrence and signal characteristics on conventional MRI of these two entities may overlap [73, 74]. ORN of the skull base occurs with a mean time of onset at 18 months upon completion of radiotherapy. Patients often present with pain, bleeding, foul odor and are typically found to have exposed and necrotic bone [76]. It may show variable MRI signals with low signal on T1-weighted images, high signal on T2-weighted fat-suppressed images and avid enhancement on postcontrast T1-weighted fat-suppressed images [77]. The identification of ORN on MRI alone is challenging and correlation with osseous findings on CT can be extremely helpful [78]. The presence of bone sclerosis, intraosseous gas and the absence of associated solid mass favors ORN rather than tumor recurrence [78]. The diagnosis of recurrent tumors is more straightforward when there are new areas of cortical destruction associated with an intermediately enhancing soft tissue mass [73]. Comparison of serial previous imaging is essential. Stable or decreasing size of bone marrow signal change or contrast enhancement favors post-treatment changes [71]. Conversely, increasing size of bone marrow signal change or contrast enhancement is highly suspicious for recurrence [72]. DWI can offer distinction between these entities. Prior work from Wang et al. has shown that the mean ADC values in clival recurrence are significantly lower than those without (0.780 ± 0.166 × 10^−3^ and 1.666 ± 0.342 × 10^−3^ mm^2^/s, respectively (*P* = 0.002)) [57]. ORN generally exhibits high ADC value unless an abscess is present from superimposed infection [77]. FDG-PET/CT may not be entirely reliable in this setting due to considerable overlap in SUV uptake between ORN and recurrent tumors leading to false positivity [77, 78].

- *#5 Familiarity with the expected imaging appearance of nasopharyngectomy is essential for accurate image interpretation of post-operative changes versus recurrent tumors.*

Salvage surgical resection in the form of nasopharyngectomy has been considered the primary treatment for residual or recurrent NPC at the primary site, as it can achieve reasonable long-term survival and better local control than reirradiation, while avoiding the toxicities of cumulative radiation injury to normal tissues [79, 80]. Nasopharyngectomy can be performed via open approaches (e.g. maxillary swing, midface degloving, transpalatal, transmaxillary, and trans-infratemporal fossa) or a transnasal endoscopic approach. The endoscopic approach has gained popularity in recent years as a minimally-invasive surgical option. In a recent meta-analysis, the endoscopic approach demonstrated higher survival rates compared to open approaches in rT1 (93% versus 87%), rT2 (77% versus 63%) and rT3 tumors (67% versus 53%), with fewer severe complications and a lower local recurrence rate (27% versus 32%) [61]. The definition of resectability has significant variation across different institutions, and the indications for nasopharyngectomy have expanded. Recurrent tumors are considered inoperable only when there is substantial intracranial extension with cavernous sinus invasion or encasement of the petrosal internal carotid artery [79, 81].

The cornerstone of successful surveillance imaging relies on familiarity with the expected changes after surgery. It is crucial to review the pretreatment imaging, as recurrent tumors are expected to exhibit similar signal characteristics as the original tumor. Furthermore, knowledge of the specific operative procedures enhances the understanding of the expected imaging changes. The common imaging appearances of open nasopharyngectomy via the maxillary swing approach and transnasal endoscopic nasopharyngectomy will be discussed.

**Fig. 13:**
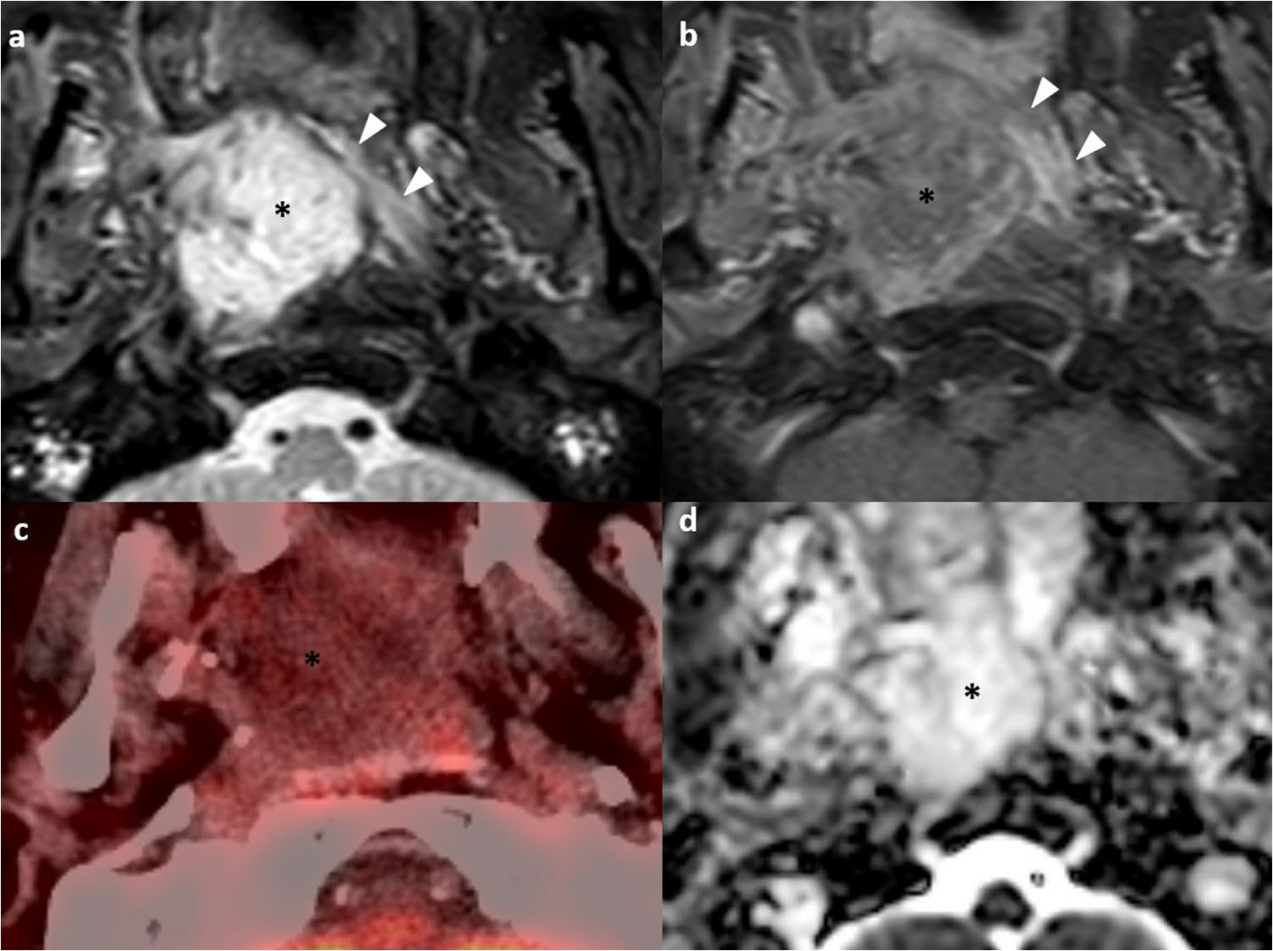
Same patient as Fig. 6. Normal expected early post-operative appearance of open nasopharyngectomy in a 72-year-old man with recurrent NPC (clinical restaging T2N0M0). He had open right nasopharyngectomy via maxillary swing approach with vastus lateralis flap followed by post-operative stereotactic radiotherapy. A follow-up MRI was performed at 2-month after the operation. Axial T2-weighted image with fat-suppression shows the flap at the right nasopharynx appearing diffusely swollen with markedly hyperintense signal (asterisk) with perifocal soft tissue edema (arrowheads) (**a**). Axial T1-weighted post-contrast image with fat suppression shows the flap with heterogenous enhancement (asterisk) and perifocal soft tissue enhancement in the operative bed (arrowheads) (**b**). The imaging appearance of the flap was confused with residual tumor and additional FDG-PET/CT as well as follow-up MRI with DWI were performed. Axial fusion FDG-PET/CT shows the flap being diffusely eumetabolic (asterisk) (**c**). Conventional sequences on follow-up MRI after a further 2 months showed no interval change in size and signal characteristics of the flap (not shown). ADC map shows that there is diffuse hyperintense signal within the flap suggestive of facilitated diffusion (asterisk) (**d**). No residual or recurrent tumor was evident

**Fig. 14:**
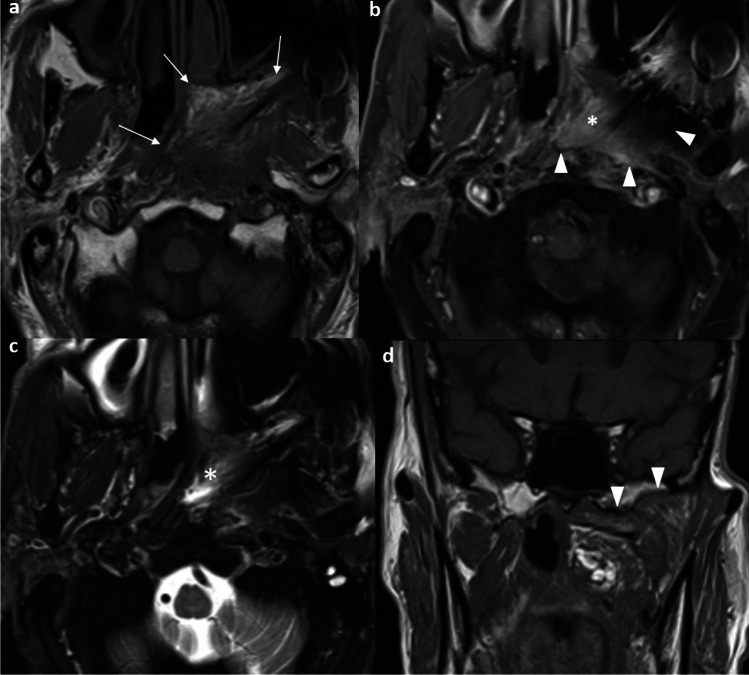
Normal expected post-operative appearance of open nasopharyngectomy in a 58-year-old man with NPC (clinical stage T4N3M0) completed chemo-irradiation in 2017. Two years after, he had a left retropharyngeal node recurrence on surveillance MRI (not shown) and subsequently an open nasopharyngectomy via maxillary swing approach and a flap reconstruction using temporalis muscle. Axial T1-weighted pre-contrast image without fat suppression one-year after the operation shows no recurrence, demonstrating normal fat striation of the temporalis muscle flap without soft tissue replacement (arrows) (**a**). Axial T1-weighted post-contrast image with fat-suppression shows non-mass like enhancement in the medial aspect of the flap (asterisk). The recipient bed of the flap shows a straight margin without discrete nodular enhancement (arrowheads) (**b**). Axial T2-weighted image with fat suppression shows mild non mass-like hyperintense signal (asterisk) (**c**). Coronal T1-weighted image without fat-suppression shows the smooth transition in the surgical bed and straight superior margin of the flap with respect to the sphenoid bone (arrowheads) (**d**)

**Fig. 15:**
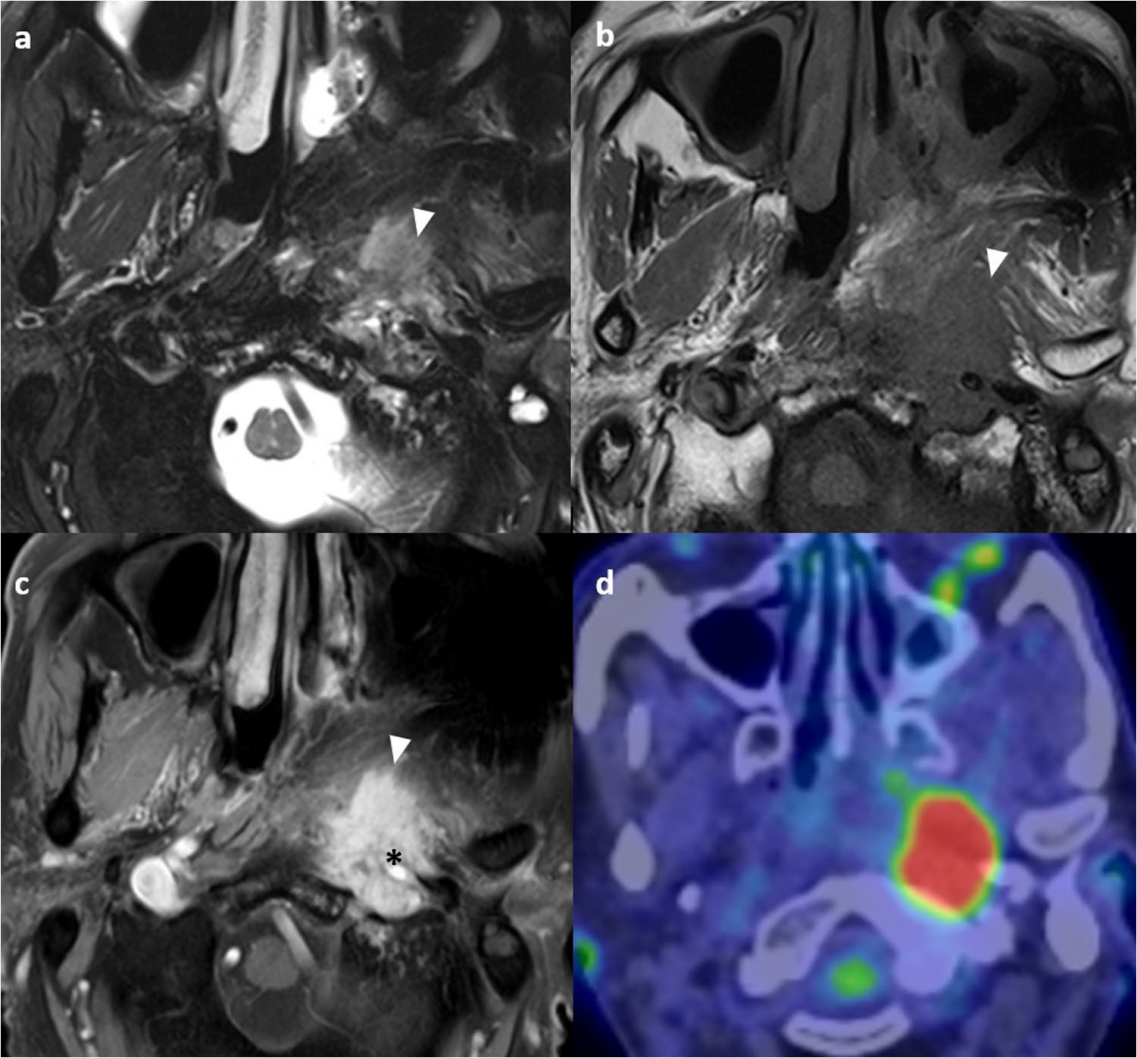
Same patient as Fig. 14. Recurrent tumor after open nasopharyngectomy in a 58-year-old man with NPC (clinical stage T4N3M0) completed chemo-irradiation in 2017 with retropharyngeal lymph node recurrence in 2019. He underwent open nasopharyngectomy via maxillary swing approach and a flap reconstruction using temporalis muscle in 2020. Axial T2-weighted image with fat suppression shows intermediate signal nodular lesion with irregular border at the recipient bed (arrowhead) (**a**). Axial T1-weighted image without fat-suppression shows the replacement of the loss of normal fatty streak of the flap with nodular soft tissue signal (arrowhead) (**b**). Axial post-contrast T1-weighted image with fat suppression shows the corresponding lesion to be intensely and homogenously enhancing (arrowhead). Encasement of ipsilateral internal carotid artery is noted indicating unresectable disease (asterisk) (**c**). Axial fusion FDG PET/CT image confirms hypermetabolism of the corresponding lesion (**d**). Overall findings are in keeping with recurrent tumor. He then received palliative chemotherapy for treatment

Open nasopharyngectomy via the maxillary swing approach involves mobilization of the maxilla to expose the nasopharynx and ipsilateral parapharyngeal space. This approach provides extensive access to the nasopharynx for tumor resection with a wide margin. With this approach, the maxilla, along with the anterior cheek tissue, is swung laterally after soft tissue dissection and osteotomies have been performed [48, 82]. There can be expected signal alterations in the operative bed early on, which show gradual normalization over time. For example, drilling of the clivus to remove the tumor involving the longus colli muscle may lead to enhancing marrow signals in the early postoperative imaging [46]. A temporalis muscle flap or vastus lateralis flap can be employed to protect exposed arteries in the maxillary osteocutaneous flap, in order to prevent carotid artery blowout, postoperative maxillary osteonecrosis, and ascending infection [60]. The flap appears more swollen with hyperintense signals on T2-weighted images, which can be related to inflammatory changes in the early postoperative period. The fatty striated appearance should be preserved. It should not show restricted diffusion on DWI nor hypermetabolism on FDG-PET/CT (Figs. 13 and 14). Over time, the T2-weighted signal and enhancement will decrease. Careful inspection of the interface between the flap and the surgical cavity (recipient bed) is important, as tumor recurrence is commonly seen at this site, where surgical or endoscopic access is difficult [83]. Smooth, non-nodular and non-mass-like enhancement can be observed in the non-fatty portion of the flap [83]. In contrast, nodularity, mass-like or focal enhancement with signal characteristics similar to those of the original tumor is a characteristic imaging appearance of recurrence [83] (Fig. 15).

**Fig. 16:**
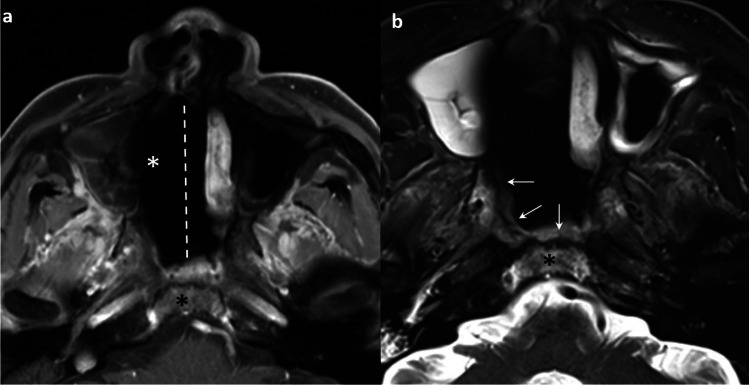
Expected post-operative changes in a 51-year-old man who had NPC (clinical T3N2M0) and completed chemoradiation in 2022. He had recurrent NPC (rT1N0M0) one-year after completion of treatment and underwent transnasal endoscopic nasopharyngectomy. A surveillance MRI was performed at 6-month post-surgery. Axial T1-weighted image with fat suppression shows the posterior septectomy of the nasal septum (dashed line) and the absence of right inferior turbinate in keeping with inferior turbinectomy (white asterisk). The clivus shows diffusely increased T2-weighted signal suggestive of marrow edema (black asterisk) (**a**). Axial T2-weighted image with fat suppression shows the nasoseptal flap covering the bony defect of the bilateral posterior wall and right lateral wall of the nasopharynx with a deeper hypointense layer and a more superficial smooth thin T2-weighted hyperintense layer (arrows). The right torus tubarius has been resected. There is also enhancing marrow edema in the clivus related to bone drilling, in keeping with post-operative change (asterisk) (**b**)

Endoscopic nasopharyngectomy is a minimally invasive approach involving endoscopic access to the nasopharynx via the nasal cavities. Different techniques exist, and the extent of resection varies with the extent of the recurrent tumor [80, 84]. MRI signal alterations in the operative bed can be expected as early as 2 weeks, being more common at the site of the recurrent tumor and its adjacent area [80]. Posterior septectomy and inferior turbinectomy can enhance exposure of the nasopharynx and surgical maneuverability [84]. Their corresponding defects should be expected on the postoperative imaging. Reconstruction via various flaps (e.g. nasoseptal flap or temporoparietal fascial flap) is needed to cover the bony defect, to avoid osteomyelitis and exposure of the internal carotid artery. Inflammatory signal change is commonly noted in the bony structures (e.g. clivus, pterygoid base) and soft tissue structures (e.g. retropharyngeal space, masticator space) in the early phase, and shows gradual resolution over time. The nasoseptal flap appears as a thin layer of tissue along the posterolateral border of the surgical field. Its mucosal layer shows homogeneous hyperintense signal on T2-weighted images and post-contrast enhancement. There should be minimal thickness reduction over time without significant signal alteration in one year [84] (Fig. 16). The temporoparietal fascial flap assumes a tri-laminated appearance with mucosal, intermediate and deep layers. It typically displays a reduction in the T2 signal and associated enhancement of the intermediate layer over time, with an increase in T1 hyperintense signal suggesting regression of inflammatory change but progressive fatty change at about one year [84].

**Fig. 17:**
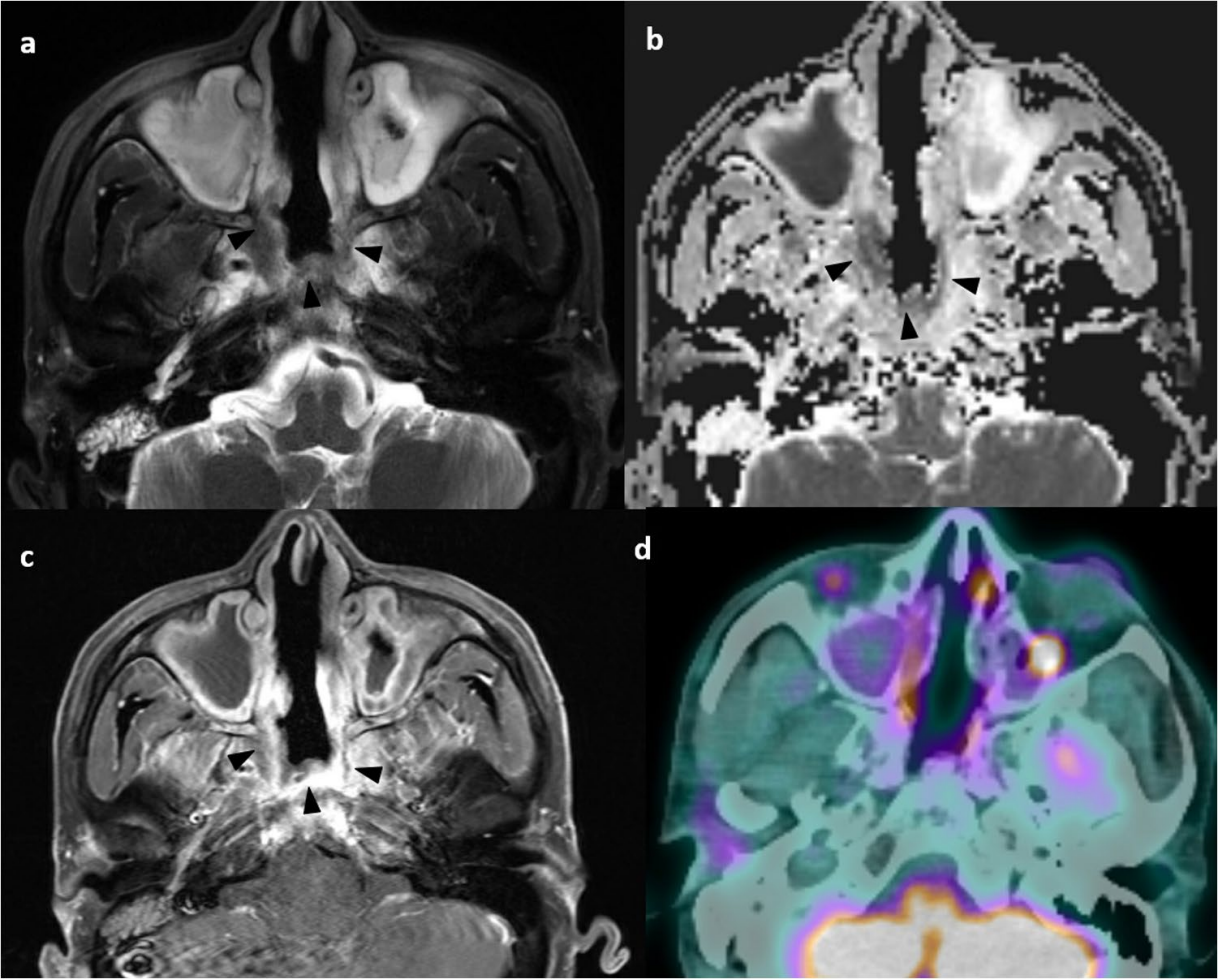
Same patient as Fig. 16. Recurrent tumor after endoscopic nasopharyngectomy in a 51-year-old man who had NPC (clinical T3N2M0) and completed chemoradiation in 2022. He had recurrent NPC (rT1N0M0) one-year after completion of treatment and underwent transnasal endoscopic nasopharyngectomy. A surveillance MRI was performed at 18-month post-surgery. Axial T2-weighted image with fat suppression at the superior resection margin shows infiltrative intermediate signal involving bilateral posterior and lateral walls of the nasopharynx (arrowheads). There is also perifocal patchy hyperintense signal involving the bilateral pterygoid bases and the clivus (**a**). ADC map shows the infiltrative soft tissue along bilateral nasopharyngeal walls to be hypointense (arrowheads) with hyperintensity on the high b-value DWI (not shown), in keeping with restricted diffusion (**b**). Axial post-contrast T1-weighted image with fat suppression shows the moderate enhancement of the soft tissue along bilateral nasopharyngeal walls (arrowheads), surrounded by marked enhancement in the adjacent soft tissue and the bone (**c**). Findings are suggestive of recurrent tumor along the nasopharyngeal walls on the background of post-operative change. Axial fusion FDG-PET/CT confirms the presence of hypermetabolic tumor along the nasopharyngeal walls. Otherwise no nodal or distant metastasis (**d**). Biopsy confirmed recurrent undifferentiated carcinoma

All these post-operative signal changes should be distinguished from the typical tumoral signal on conventional MRI. DWI and FDG-PET/CT are problem-solving tools in diagnostic dilemmas, and the presence of restricted diffusion on DWI and hypermetabolism on FDG-PET/CT will increase confidence in diagnosing recurrent tumors (Fig. 17).

## Artificial intelligence (AI) in NPC imaging

There is growing interest in using AI for the clinical management of NPC, particularly through deep learning models that leverage imaging data for diagnosis, radiotherapy planning and prognosis prediction. It aims to enhance work efficiency, diagnostic accuracy and promote personalized treatment. Recent systematic reviews show most published researches are in areas of automatic segmentation for radiotherapy planning and prognosis prediction. Currently the use of AI is primarily limited to specific task performances in the clinical workflow. For radiotherapy planning, AI aids in automating the contouring of primary tumors and organ at risk on imaging, thereby enhancing the accuracy and work efficiency of radiation oncologists [85]. Additionally, AI models help predict patient outcomes (e.g. survival, response to treatment) based on imaging data. However, there are still conflicting results with regards to the diagnostic capacity of AI (e.g. tumor detection) compared to humans due to varying sizes, complexities of tumors, as well as differing reader experience affecting result comparison [86, 87]. For image acquisition, a recent study found that AI assisted compressed sensing technique not only reduced examination time, but also improved image quality [88]. Despite these advancements, barriers remain for the robust application of AI in clinical setting, including a lack of large-scale labelled dataset, standardization of scanning protocols, data privacy concerns and limited generalizability [87].

## Conclusion

Imaging is essential in precision oncological management in NPC. A good understanding of imaging pearls and pitfalls helps radiologists in stepping forward to provide reliable guidance for clinicians and improvement in patients’ health outcome.

## Data Availability

No datasets were generated or analysed during the current study.

## References

[1] Zhang Y, Rumgay H, Li M, Cao S, Chen W (2023) Nasopharyngeal cancer incidence and mortality in 185 countries in 2020 and the projected burden in 2040: population-based global epidemiological profiling. JMIR Public Health Surveill 20(9):e49968. 10.2196/49968 10.2196/49968 PMC10551785 37728964

[2] King AD (2022) MR imaging of nasopharyngeal carcinoma. Magn Reson Imaging Clin N Am 30(1):19–33. 10.1016/j.mric.2021.06.015 34802578 10.1016/j.mric.2021.06.015

[3] Ng WT, Chow JCH, Beitler JJ, Corry J, Mendenhall W, Lee AWM, Robbins KT, Nuyts S, Saba NF, Smee R, Stokes WA, Strojan P, Ferlito A (2022) Current radiotherapy considerations for nasopharyngeal carcinoma. Cancers (Basel) 14(23):5773. 10.3390/cancers14235773 36497254 10.3390/cancers14235773 PMC9736118

[4] Li WF, Sun Y, Chen M, Tang LL, Liu LZ, Mao YP, Chen L, Zhou GQ, Li L, Ma J (2012) Locoregional extension patterns of nasopharyngeal carcinoma and suggestions for clinical target volume delineation. Chin J Cancer 31(12):579–587. 10.5732/cjc.012.10095 22854064 10.5732/cjc.012.10095 PMC3777458

[5] Liang SB, Sun Y, Liu LZ, Chen Y, Chen L, Mao YP, Tang LL, Tian L, Lin AH, Liu MZ, Li L, Ma J (2009) Extension of local disease in nasopharyngeal carcinoma detected by magnetic resonance imaging: improvement of clinical target volume delineation. Int J Radiat Oncol Biol Phys 75(3):742–750. 10.1016/j.ijrobp.2008.11.053 19251378 10.1016/j.ijrobp.2008.11.053

[6] Dubrulle F, Souillard R, Hermans R (2007) Extension patterns of nasopharyngeal carcinoma. Eur Radiol 17(10):2622–2630. 10.1007/s00330-007-0616-z 17404741 10.1007/s00330-007-0616-z

[7] Lee AW, Ng WT, Pan JJ, Poh SS, Ahn YC, AlHussain H, Corry J, Grau C, Grégoire V, Harrington KJ, Hu CS, Kwong DL, Langendijk JA, Le QT, Lee NY, Lin JC, Lu TX, Mendenhall WM, O’Sullivan B, Ozyar E, Peters LJ, Rosenthal DI, Soong YL, Tao Y, Yom SS, Wee JT (2018) International guideline for the delineation of the clinical target volumes (CTV) for nasopharyngeal carcinoma. Radiother Oncol 126(1):25–36. 10.1016/j.radonc.2017.10.032 29153464 10.1016/j.radonc.2017.10.032

[8] Ho FC, Tham IW, Earnest A, Lee KM, Lu JJ (2012) Patterns of regional lymph node metastasis of nasopharyngeal carcinoma: a meta-analysis of clinical evidence. BMC Cancer 21(12):98. 10.1186/1471-2407-12-98 10.1186/1471-2407-12-98 PMC3353248 22433671

[9] Song C, Cheng P, Cheng J, Zhang Y, Xie S (2021) Value of apparent diffusion coefficient histogram analysis in the differential diagnosis of nasopharyngeal lymphoma and nasopharyngeal carcinoma based on readout-segmented diffusion-weighted imaging. Front Oncol 12(11):632796. 10.3389/fonc.2021.632796 10.3389/fonc.2021.632796 PMC7996088 33777787

[10] Huang Z, Liao K (2023) A review of the role of DWI in radiation therapy planning and treatment response assessment for NPC management. Curr Res Med Sci 2:84–90. 10.56397/CRMS.2023.03.13

[11] Chan KCA, Woo JKS, King A, Zee BCY, Lam WKJ, Chan SL, Chu SWI, Mak C, Tse IOL, Leung SYM, Chan G, Hui EP, Ma BBY, Chiu RWK, Leung SF, van Hasselt AC, Chan ATC, Lo YMD (2017) Analysis of plasma epstein-barr virus DNA to screen for nasopharyngeal cancer. N Engl J Med 377(6):513–522. 10.1056/NEJMoa1701717 28792880 10.1056/NEJMoa1701717

[12] King AD, Woo JKS, Ai QY, Chan JSM, Lam WKJ, Tse IOL, Bhatia KS, Zee BCY, Hui EP, Ma BBY, Chiu RWK, van Hasselt AC, Chan ATC, Lo YMD, Chan KCA (2019) Complementary roles of MRI and endoscopic examination in the early detection of nasopharyngeal carcinoma. Ann Oncol 30(6):977–982. 10.1093/annonc/mdz106 30912815 10.1093/annonc/mdz106

[13] Gorolay VV, Niles NN, Huo YR, Ahmadi N, Hanneman K, Thompson E, Chan MV (2022) MRI detection of suspected nasopharyngeal carcinoma: a systematic review and meta-analysis. Neuroradiology 64(8):1471–1481. 10.1007/s00234-022-02941-w 35499636 10.1007/s00234-022-02941-w PMC9271105

[14] Pastor M, Lopez Pousa A, Del Barco E, Perez Segura P, Astorga BG, Castelo B, Bonfill T, Martinez Trufero J, Grau JJ, Mesia R (2018) SEOM clinical guideline in nasopharynx cancer (2017). Clin Transl Oncol 20(1):84–88. 10.1007/s12094-017-1777-0 29098554 10.1007/s12094-017-1777-0 PMC5785612

[15] King AD, Ai QYH, Lam WKJ, Tse IOL, So TY, Wong LM, Tsang JYM, Leung HS, Zee BCY, Hui EP, Ma BBY, Vlantis AC, van Hasselt AC, Chan ATC, Woo JKS, Chan KCA (2024) Early detection of nasopharyngeal carcinoma: performance of a short contrast-free screening magnetic resonance imaging. J Natl Cancer Inst 116(5):665–672. 10.1093/jnci/djad260 38171488 10.1093/jnci/djad260

[16] Bage AM, Karthikeyan AD, Bage NN (2014) Adenoid hypertrophy existence in adulthood- a truth revealed. Natl J Clin Anat 3(1):12–16. 10.4103/2277-4025.297360

[17] Hoffmann TK, Hahn J (2025) Nasopharyngeal masses in adults—A retrospective analysis of 255 patients to evaluate symptoms, clinical findings, and histological results. World J Otorhinolaryngol Head Neck Surg 11(01):45–51. 10.1002/wjo2.139 40070493 10.1002/wjo2.139 PMC11891276

[18] Bhatia KS, King AD, Vlantis AC, Ahuja AT, Tse GM (2012) Nasopharyngeal mucosa and adenoids: appearance at MR imaging. Radiology 263(2):437–443. 10.1148/radiol.12111349 22403169 10.1148/radiol.12111349

[19] King AD, Wong LYS, Law BKH, Bhatia KS, Woo JKS, Ai QY, Tan TY, Goh J, Chuah KL, Mo FKF, Chan KCA, Chan ATC, Vlantis AC (2018) MR imaging criteria for the detection of nasopharyngeal carcinoma: discrimination of early-stage primary tumors from benign hyperplasia. AJNR Am J Neuroradiol 39(3):515–523. 10.3174/ajnr.A5493 29284600 10.3174/ajnr.A5493 PMC7655321

[20] Wu YP, Cai PQ, Tian L, Xu JH, Mitteer RA Jr, Fan Y, Zhang Z (2015) Hypertrophic adenoids in patients with nasopharyngeal carcinoma: appearance at magnetic resonance imaging before and after treatment. Chin J Cancer 34(3):130–136. 10.1186/s40880-015-0005-y 25962737 10.1186/s40880-015-0005-y PMC4593340

[21] Johnston M, Yu E, Kim J (2012) Perineural invasion and spread in head and neck cancer. Expert Rev Anticancer Ther 12(3):359–371. 10.1586/era.12.9 22369327 10.1586/era.12.9

[22] Schmitd LB, Scanlon CS, D’Silva NJ (2018) Perineural invasion in head and neck cancer. J Dent Res 97(7):742–750. 10.1177/0022034518756297 29443582 10.1177/0022034518756297 PMC6728584

[23] Brown IS (2016) Pathology of perineural spread. J Neurol Surg B Skull Base 77(2):124–130. 10.1055/s-0036-1571837 27123388 10.1055/s-0036-1571837 PMC4846404

[24] Liu X, Liu LZ, Mao YP, Chen L, Tang LL, Zhou GQ, Sun Y, Yue D, Lin AH, Li L, Ma J (2014) Prognostic value of magnetic resonance imaging-detected cranial nerve invasion in nasopharyngeal carcinoma. Br J Cancer 110(6):1465–1471. 10.1038/bjc.2014.27 24496459 10.1038/bjc.2014.27 PMC3960608

[25] Lee H, Lazor JW, Assadsangabi R, Shah J (2019) An imager’s guide to perineural tumor spread in head and neck cancers: radiologic footprints on ^18^F-FDG PET, with CT and MRI correlates. J Nucl Med 60(3):304–311. 10.2967/jnumed.118.214312 30291196 10.2967/jnumed.118.214312

[26] Nie X, Zhou J, Zeng J, Sun J, Chen W, Niu J (2022) Does PET scan have any role in the diagnosis of perineural spread associated with the head and neck tumors? Adv Clin Exp Med 31(8):827–835. 10.17219/acem/14735935467086 10.17219/acem/147359

[27] Schroeder C, Lee JH, Tetzner U, Seidel S, Kim SY (2020) Comparison of diffusion-weighted MR imaging and ^18^F Fluorodeoxyglucose PET/CT in detection of residual or recurrent tumors and delineation of their local spread after (chemo) radiotherapy for head and neck squamous cell carcinoma. Eur J Radiol 130:109157. 10.1016/j.ejrad.2020.109157 32652403 10.1016/j.ejrad.2020.109157

[28] National Comprehensive Cancer Network (2024) NCCN clinical practice guidelines in oncology: head and neck cancers (version 4.2024) [PDF]. https://www.nccn.org/guidelines/guidelines-detail?category=1&id=1437 10.6004/jnccn.2020.0031 32634781

[29] Lin C, Lu N, Liang JL, Guo J, Gu LW, Sun R, Guo L, Yang Q (2023) Clinical treatment considerations in the intensity-modulated radiotherapy era for parotid lymph node metastasis in patients with nasopharyngeal carcinoma. Radiother Oncol 186:109802. 10.1016/j.radonc.2023.109802 37423477 10.1016/j.radonc.2023.109802

[30] Zhang Y, Zhang ZC, Li WF, Liu X, Liu Q, Ma J (2019) Prognosis and staging of parotid lymph node metastasis in nasopharyngeal carcinoma: An analysis in 10,126 patients. Oral Oncol 95:150–156. 10.1016/j.oraloncology.2019.06.013 31345383 10.1016/j.oraloncology.2019.06.013

[31] Lan M, Huang Y, Chen CY, Han F, Wu SX, Tian L, Zheng L, Lu TX (2015) Prognostic value of cervical nodal necrosis in nasopharyngeal carcinoma: analysis of 1800 patients with positive cervical nodal metastasis at MR imaging. Radiology 276(2):536–544. 10.1148/radiol.15141251 25759968 10.1148/radiol.15141251

[32] Ai QH, Hung KF, So TY, Mo FKF, Tsung Anthony Chin W, Hui EP, Ma BBY, Ying M, King AD (2022) Prognostic value of cervical nodal necrosis on staging imaging of nasopharyngeal carcinoma in era of intensity-modulated radiotherapy: a systematic review and meta-analysis. Cancer Imaging 22(1):24. 10.1186/s40644-022-00462-6 35596198 10.1186/s40644-022-00462-6 PMC9123677

[33] Huang SH, Chernock R, O’Sullivan B, Fakhry C (2021) Assessment criteria and clinical implications of extranodal extension in head and neck cancer. Am Soc Clin Oncol Educ Book 41:265–278. 10.1200/EDBK_320939 34010048 10.1200/EDBK_320939

[34] Ai QY, King AD, Poon DMC, Mo FKF, Hui EP, Tong M, Ahuja AT, Ma BBY, Chan ATC (2019) Extranodal extension is a criterion for poor outcome in patients with metastatic nodes from cancer of the nasopharynx. Oral Oncol 88:124–130. 10.1016/j.oraloncology.2018.11.007 30616782 10.1016/j.oraloncology.2018.11.007

[35] Henson C, Abou-Foul AK, Yu E, Glastonbury C, Huang SH, King AD, Lydiatt WM, McDowell L, Nagelschneider AA, Nankivell PC, O’Sullivan B, Rhys R, Xiao Y, Andrew D, Asmussen JT, Bidault F, Dankbaar JW, de Graaf P, Gebrim ES, Hu C, Ding J, Kanda T, Kim J, Kuno H, Medrano-Martorell S, Oikonomopoulos N, Goh JP, Santos-Armentia E, Schafigh DG, Subramaniam RM, Wu XC, Yom SS, Mehanna H (2024) Criteria for the diagnosis of extranodal extension detected on radiological imaging in head and neck cancer: Head and Neck Cancer International Group consensus recommendations. Lancet Oncol 25(7):e297–e307. 10.1016/S1470-2045(24)00066-4 38936388 10.1016/S1470-2045(24)00066-4

[36] Ai QYH, King AD, Yuan H, Vardhanabhuti V, Mo FKF, Hung KF, Hui EP, Kwong DL, Lee VH, Ma BBY (2024) Radiologic extranodal extension for nodal staging in nasopharyngeal carcinoma. Radiother Oncol 191:110050. 10.1016/j.radonc.2023.110050 38101457 10.1016/j.radonc.2023.110050

[37] Pan JJ, Mai HQ, Ng WT, Hu CS, Li JG, Chen XZ, Chow JCH, Wong E, Lee V, Ma LY, Guo QJ, Liu Q, Liu LZ, Xu TT, Gong XC, Qiang MY, Au KH, Liu TC, Chiang CL, Xiao YP, Lin SJ, Chen YB, Guo SS, Wong CHL, Tang LQ, Xu ZY, Jia YZ, Peng WS, Hu LP, Lu TZ, Jiang F, Cao CN, Xu W, Ma J, Blanchard P, Williams M, Glastonbury CM, King AD, Patel SG, Seethala RR, Colevas AD, Fan DM, Chua MLK, Huang SH, O’Sullivan B, Lydiatt W, Lee AWM (2024) Ninth version of the AJCC and UICC nasopharyngeal cancer TNM staging classification. JAMA Oncol 10(12):1627–1635. 10.1001/jamaoncol.2024.4354 39388190 10.1001/jamaoncol.2024.4354 PMC11581663

[38] Wong KY, Wong KC (2021) Diagnostic dilemma between skull base osteomyelitis and nasopharyngeal carcinoma: a case series. Hong Kong Med J 27(4):300–302. 10.12809/hkmj208555 34413260 10.12809/hkmj208555

[39] Lo ES, Kwok HM, Pan NY (2024) Imaging spectrum and complications of otogenic infections: insights into delayed diagnosis. Br J Radiol 97(1156):726–733. 10.1093/bjr/tqae015 38335140 10.1093/bjr/tqae015 PMC11027324

[40] Chapman PR, Choudhary G, Singhal A (2021) Skull base osteomyelitis: a comprehensive imaging review. AJNR Am J Neuroradiol 42(3):404–413. 10.3174/ajnr.A7015 33478944 10.3174/ajnr.A7015 PMC7959418

[41] Álvarez Jáñez F, Barriga LQ, Iñigo TR, Roldán Lora F (2021) Diagnosis of skull base osteomyelitis. Radiographics 41(1):156–174. 10.1148/rg.2021200046 33411616 10.1148/rg.2021200046

[42] Kwok HM, Ng FH, Chau CM, Lam SY, Ma JKF (2022) Multimodality imaging of extra-nodal lymphoma in the head and neck. Clin Radiol 77(8):e549–e559. 10.1016/j.crad.2022.04.017 35641340 10.1016/j.crad.2022.04.017

[43] Tabnak P, HajiEsmailPoor Z (2023) Differentiating nasopharyngeal carcinoma from lymphoma in the head and neck region using the apparent diffusion coefficient (ADC) value: a systematic review and meta-analysis. Pol J Radiol 17(88):e472–e482. 10.5114/pjr.2023.132172 10.5114/pjr.2023.132172 PMC10660142 38020498

[44] Chen S, Yang D, Liao X, Lu Y, Yu B, Xu M, Bin Y, Zhou P, Yang Z, Liu K, Wang R, Zhao T, Kang M (2022) Failure patterns of recurrence and metastasis after intensity-modulated radiotherapy in patients with nasopharyngeal carcinoma: results of a multicentric clinical study. Front Oncol 11(11):693199. 10.3389/fonc.2021.693199 35223448 10.3389/fonc.2021.693199 PMC8874804

[45] Xiao XT, Wu YS, Chen YP, Liu X, Guo R, Tang LL, Ma J, Li WF (2023) Patterns and prognosis of regional recurrence in nasopharyngeal carcinoma after intensity-modulated radiotherapy. Cancer Med 12(2):1399–1408. 10.1002/cam4.5020 35822664 10.1002/cam4.5020 PMC9883543

[46] Teo PT, Tan NC, Khoo JB (2013) Imaging appearances for recurrent nasopharyngeal carcinoma and post-salvage nasopharyngectomy. Clin Radiol 68(11):e629–e638. 10.1016/j.crad.2013.06.003 23937825 10.1016/j.crad.2013.06.003

[47] Meng K, Tey J, Ho FCH, Asim H, Cheo T (2020) Utility of magnetic resonance imaging in determining treatment response and local recurrence in nasopharyngeal carcinoma treated curatively. BMC Cancer 20(1):193. 10.1186/s12885-020-6664-3 32143592 10.1186/s12885-020-6664-3 PMC7060635

[48] Lee AW, Foo W, Law SC, Poon YF, Sze WM, Tung SY, Chappell R, Lau WH, Ho JH (1999) Recurrent nasopharyngeal carcinoma: the puzzles of long latency. Int J Radiat Oncol Biol Phys 44(1):149–56. 10.1016/s0360-3016(98)00524-0 10219808 10.1016/s0360-3016(98)00524-0

[49] Chan AT, Grégoire V, Lefebvre JL, Licitra L, Felip E, EHNS-ESMO-ESTRO Guidelines Working Group (2010) Nasopharyngeal cancer: EHNS-ESMO-ESTRO Clinical Practice Guidelines for diagnosis, treatment and follow-up. Ann Oncol 21(Suppl 5):v187-9. 10.1093/annonc/mdq186 20555078 10.1093/annonc/mdq186

[50] Kwong DL, Nicholls J, Wei WI, Chua DT, Sham JS, Yuen PW et al (1999) The time course of histologic remission after treatment of patients with nasopharyngeal carcinoma. Cancer 85(7):1446–1453 10193933 10.1002/(sici)1097-0142(19990401)85:7<1446::aid-cncr4>3.0.co;2-3

[51] Li WF, Zhang Y, Liu X, Tang LL, Tian L, Guo R et al (2017) Delayed clinical complete response to intensity-modulated radiotherapy in nasopharyngeal carcinoma. Oral Oncol 75:120–126 29224808 10.1016/j.oraloncology.2017.10.020

[52] Zuchowski C, Kemme J, Aiken AH, Baugnon KL, Abdel Razek AAK, Wu X (2022) Posttreatment magnetic resonance imaging surveillance of head and neck cancers. Magn Reson Imaging Clin N Am 30(1):109–120. 10.1016/j.mric.2021.06.018 34802574 10.1016/j.mric.2021.06.018

[53] Lee CC, Lee JC, Huang WY, Juan CJ, Jen YM, Lin LF (2021) Image-based diagnosis of residual or recurrent nasopharyngeal carcinoma may be a phantom tumor phenomenon. Medicine (Baltimore) 100(8):e24555. 10.1097/MD.0000000000024555 33663063 10.1097/MD.0000000000024555 PMC7909123

[54] Ng SH, Liu HM, Ko SF, Hao SP, Chong VF (2002) Posttreatment imaging of the nasopharynx. Eur J Radiol 44(2):82–95. 10.1016/s0720-048x(02)00061-x 12413677 10.1016/s0720-048x(02)00061-x

[55] Ahuja CK, Agarwal V, Jain C, Vyas S, Kumar J, Singh P (2023) Imaging recommendations for diagnosis, staging, and management of nasopharynx carcinoma. Indian J Med Paediatr Oncol 44:175–180. 10.1055/s-0042-1760309

[56] So TY, Vardhanabhuti V (2021) Essential imaging of the nasopharyngeal space with special focus on nasopharyngeal carcinoma. Oper Tech Otolaryngol Head Neck Surg 32(1):8–14. 10.1016/j.otot.2021.01.002. (ISSN 1043-1810)

[57] Wang C, Liu L, Lai S, Su D, Liu Y, Jin G, Zhu X, Luo N (2018) Diagnostic value of diffusion-weighted magnetic resonance imaging for local and skull base recurrence of nasopharyngeal carcinoma after radiotherapy. Medicine (Baltimore) 97(34):e11929. 10.1097/MD.0000000000011929 30142809 10.1097/MD.0000000000011929 PMC6112862

[58] Comoretto M, Balestreri L, Borsatti E, Cimitan M, Franchin G, Lise M (2008) Detection and restaging of residual and/or recurrent nasopharyngeal carcinoma after chemotherapy and radiation therapy: comparison of MR imaging and FDG PET/CT. Radiology 249(1):203–211. 10.1148/radiol.2491071753 18710963 10.1148/radiol.2491071753

[59] Jung JH, Choi Y, Im KC (2016) PET/MRI: Technical challenges and recent advances. Nucl Med Mol Imaging 50(1):3–12. 10.1007/s13139-016-0393-1 26941854 10.1007/s13139-016-0393-1 PMC4762872

[60] Ding XI, Liu Y-P, Hua Y-J, Zou X, Wang Z-Q, Xie Y-L, Chen M-Y (2021) Clinical advances in nasopharyngeal carcinoma surgery and a video demonstration. Vis Cancer Med 2:2

[61] Li G, Wang J, Tang H, Han R, Zhao Y, Wang X, Zhou H (2020) Comparing endoscopic surgeries with open surgeries in terms of effectiveness and safety in salvaging residual or recurrent nasopharyngeal cancer: Systematic review and meta-analysis. Head Neck 42(11):3415–3426. 10.1002/hed.26397 33463833 10.1002/hed.26397

[62] Hua YJ, Chen MY, Qian CN, Hong MH, Zhao C, Guo L, Guo X, Cao KJ (2009) Postradiation nasopharyngeal necrosis in the patients with nasopharyngeal carcinoma. Head Neck 31(6):807–812. 10.1002/hed.21036 19340873 10.1002/hed.21036

[63] Tang J, Li XW, Wu Y, Su Z, He Y, Sun XW, Cao XL, Li YH, Wang BC, Zou GR (2023) Treating radiation-related nasopharyngeal necrosis with endostar in patient with nasopharyngeal carcinoma: A report of two cases and a literature review. Mol Clin Oncol 19(1):57. 10.3892/mco.2023.2653 37359714 10.3892/mco.2023.2653 PMC10288433

[64] Chen MY, Mai HQ, Sun R, Guo X, Zhao C, Hong MH, Hua YJ (2013) Clinical findings and imaging features of 67 nasopharyngeal carcinoma patients with postradiation nasopharyngeal necrosis. Chin J Cancer 32(10):533–538. 10.5732/cjc.012.10252 23816556 10.5732/cjc.012.10252 PMC3845539

[65] Li KY, Kwok HM, Cheuk W, Ma KFJ (2024) Demystifying the challenging diagnosis of post-radiation nasopharyngeal necrosis on multimodality imaging. J Med Imaging Radiat Oncol 68(7):805–807. 10.1111/1754-9485.13784 39315669 10.1111/1754-9485.13784

[66] Loke SC, Karandikar A, Ravanelli M, Farina D, Goh JP, Ling EA, Maroldi R, Tan TY (2016) Superior cervical ganglion mimicking retropharyngeal adenopathy in head and neck cancer patients: MRI features with anatomic, histologic, and surgical correlation. Neuroradiology 58(1):45–50. 10.1007/s00234-015-1598-1 26423907 10.1007/s00234-015-1598-1

[67] Subodh A, Bhatt AA (2023) Hypertrophied superior cervical ganglia after radiotherapy for head and neck cancer. AJR Am J Roentgenol 221(5):695. 10.2214/AJR.23.29790 37466192 10.2214/AJR.23.29790

[68] Cho SJ, Lee JH, Park JE, Choi YJ, Kim JH, Kim HJ, Baek JH (2018) Serial magnetic resonance imaging evaluations of irradiated superior cervical sympathetic ganglia: Not every retropharyngeal enlarging mass is a sign of malignancy. Eur J Radiol 98:126–129. 10.1016/j.ejrad.2017.11.008 29279150 10.1016/j.ejrad.2017.11.008

[69] Lee JY, Lee JH, Song JS, Song MJ, Hwang SJ, Yoon RG, Jang SW, Park JE, Heo YJ, Choi YJ, Baek JH (2016) Superior cervical sympathetic ganglion: normal imaging appearance on 3T-MRI. Korean J Radiol. 17(5):657–63. 10.3348/kjr.2016.17.5.657 27587954 10.3348/kjr.2016.17.5.657 PMC5007392

[70] Ravanelli M, Tononcelli E, Leali M, Buffa I, Loke SC, Karandikar A, Chokkapan K, Yue GOC, Goh JPN, Tan TY, Farina D (2020) Magnetic resonance imaging features of the superior cervical ganglion and expected changes after radiation therapy to the head and neck in a long-term follow-up. Neuroradiology 62(4):519–524. 10.1007/s00234-020-02373-4 31996966 10.1007/s00234-020-02373-4

[71] Yokota H, Mukai H, Hattori S, Yamada K, Anzai Y, Uno T (2018) MR Imaging of the superior cervical ganglion and inferior ganglion of the vagus nerve: structures that can mimic pathologic retropharyngeal lymph nodes. AJNR Am J Neuroradiol 39(1):170–176. 10.3174/ajnr.A5434 29122764 10.3174/ajnr.A5434 PMC7410715

[72] Parlak S, Yazici G, Dolgun A, Ozgen B (2021) The evolution of bone marrow signal changes at the skull base in nasopharyngeal carcinoma patients treated with radiation therapy. Radiol Med 126(6):818–826. 10.1007/s11547-021-01342-y 33788155 10.1007/s11547-021-01342-y

[73] King AD, Ahuja AT, Yeung DK, Wong JK, Lee YY, Lam WW, Ho SS, Yu SC, Leung SF (2007) Delayed complications of radiotherapy treatment for nasopharyngeal carcinoma: imaging findings. Clin Radiol 62(3):195–203. 10.1016/j.crad.2006.10.011 17293211 10.1016/j.crad.2006.10.011

[74] Li JX, Lu TX, Huang Y, Han F (2012) Clinical characteristics of recurrent nasopharyngeal carcinoma in high-incidence area. ScientificWorldJournal 2012:719754. 10.1100/2012/719754 22448138 10.1100/2012/719754 PMC3289855

[75] Lam WKJ, Chan KCA, Lo YMD (2019) Plasma epstein-barr virus DNA as an archetypal circulating tumour DNA marker. J Pathol 247(5):641–649. 10.1002/path.5249 30714167 10.1002/path.5249 PMC6594142

[76] Leonetti JP, Weishaar JR, Gannon D, Harmon GA, Block A, Anderson DE (2020) Osteoradionecrosis of the skull base. J Neurooncol 150(3):477–482. 10.1007/s11060-020-03462-3 32394326 10.1007/s11060-020-03462-3

[77] Varoquaux A, Rager O, Dulguerov P, Burkhardt K, Ailianou A, Becker M (2015) Diffusion-weighted and PET/MR imaging after radiation therapy for malignant head and neck tumors. Radiographics 35(5):1502–27. 10.1148/rg.2015140029 26252192 10.1148/rg.2015140029

[78] Alhilali L, Reynolds AR, Fakhran S (2014) Osteoradionecrosis after radiation therapy for head and neck cancer: differentiation from recurrent disease with CT and PET/CT imaging. AJNR Am J Neuroradiol 35(7):1405–1411. 10.3174/ajnr.A3879 24627451 10.3174/ajnr.A3879 PMC7966581

[79] Lam WKJ, Chan JYK (2018) Recent advances in the management of nasopharyngeal carcinoma. F1000Res 7:1829 10.12688/f1000research.15066.1 PMC6249636 30519454

[80] Liu Q, Sun X, Li H, Zhou J, Gu Y, Zhao W, Li H, Yu H, Wang D (2021) Types of transnasal endoscopic nasopharyngectomy for recurrent nasopharyngeal carcinoma: Shanghai EENT hospital experience. Front Oncol 10:555862. 10.3389/fonc.2020.555862 33585184 10.3389/fonc.2020.555862 PMC7873878

[81] Li H, Wang DL, Liu XW, Chen MY, Mo YX, Geng ZJ, Xie CM (2013) MRI signal changes in the skull base bone after endoscopic nasopharyngectomy for recurrent NPC: a serial study of 9 patients. Eur J Radiol 82(2):309–315. 10.1016/j.ejrad.2012.10.022 23177186 10.1016/j.ejrad.2012.10.022

[82] Wei WI, Lam KH, Sham JS (1991) New approach to the nasopharynx: the maxillary swing approach. Head Neck 13(3):200–7. 10.1002/hed.2880130306 2037471 10.1002/hed.2880130306

[83] McCarty JL, Corey AS, El-Deiry MW, Baddour HM, Cavazuti BM, Hudgins PA (2019) Imaging of Surgical Free Flaps in Head and Neck Reconstruction. AJNR Am J Neuroradiol 40(1):5–13. 10.3174/ajnr.A5776 30409846 10.3174/ajnr.A5776 PMC7048589

[84] Rondi P, Ravanelli M, Rampinelli V, Hussain IZ, Ramanzin M, Di Meo N, Borghesi A, Tomasoni M, Schreiber A, Mattavelli D, Piazza C, Farina D (2024) Magnetic resonance imaging after nasopharyngeal endoscopic resection and skull base reconstruction. J Clin Med 13(9):2624. 10.3390/jcm13092624 38731151 10.3390/jcm13092624 PMC11084522

[85] Lin L, Dou Q, Jin YM, Zhou GQ, Tang YQ, Chen WL, Su BA, Liu F, Tao CJ, Jiang N, Li JY, Tang LL, Xie CM, Huang SM, Ma J, Heng PA, Wee JTS, Chua MLK, Chen H, Sun Y (2019) Deep learning for automated contouring of primary tumor volumes by MRI for nasopharyngeal carcinoma. Radiology 291(3):677–686. 10.1148/radiol.2019182012 30912722 10.1148/radiol.2019182012

[86] Yang X, Wu J, Chen X (2023) Application of artificial intelligence to the diagnosis and therapy of nasopharyngeal carcinoma. J Clin Med 12(9):3077. 10.3390/jcm12093077 37176518 10.3390/jcm12093077 PMC10178972

[87] Ng WT, But B, Choi HCW, de Bree R, Lee AWM, Lee VHF, López F, Mäkitie AA, Rodrigo JP, Saba NF, Tsang RKY, Ferlito A (2022) Application of artificial intelligence for nasopharyngeal carcinoma management - a systematic review. Cancer Manag Res 26(14):339–366. 10.2147/CMAR.S341583 10.2147/CMAR.S341583 PMC8801370 35115832

[88] Liu H, Deng D, Zeng W, Huang Y, Zheng C, Li X, Li H, Xie C, He H, Xu G (2023) AI-assisted compressed sensing and parallel imaging sequences for MRI of patients with nasopharyngeal carcinoma: comparison of their capabilities in terms of examination time and image quality. Eur Radiol 33(11):7686–7696. 10.1007/s00330-023-09742-6 37219618 10.1007/s00330-023-09742-6 PMC10598173

